# Investigating G-protein coupled receptor signalling with light-emitting biosensors

**DOI:** 10.3389/fphys.2023.1310197

**Published:** 2024-01-08

**Authors:** Alexander Demby, Manuela Zaccolo

**Affiliations:** Department of Physiology, Anatomy and Genetics, University of Oxford, Oxford, United Kingdom

**Keywords:** signalling, GPCR, G protein, fluorescent biosensor, FRET, BRET, bioluminescence

## Abstract

G protein-coupled receptors (GPCRs) are the most frequent target of currently approved drugs and play a central role in both physiological and pathophysiological processes. Beyond the canonical understanding of GPCR signal transduction, the importance of receptor conformation, beta-arrestin (β-arr) biased signalling, and signalling from intracellular locations other than the plasma membrane is becoming more apparent, along with the tight spatiotemporal compartmentalisation of downstream signals. Fluorescent and bioluminescent biosensors have played a pivotal role in elucidating GPCR signalling events in live cells. To understand the mechanisms of action of the GPCR-targeted drugs currently available, and to develop new and better GPCR-targeted therapeutics, understanding these novel aspects of GPCR signalling is critical. In this review, we present some of the tools available to interrogate each of these features of GPCR signalling, we illustrate some of the key findings which have been made possible by these tools and we discuss their limitations and possible developments.

## 1 Introduction

G-protein coupled receptors (GPCRs) constitute the largest family of cell-surface receptors. They regulate a myriad of physiological processes in response to a large spectrum of chemical ligands, including hormones, neurotransmitters, lipids, and odorants and non-chemical stimuli including light and mechanical stress (Pierce et al., 2002). As a result of their diverse roles, GPCRs are involved in the pathophysiology of many diseases (Heng et al., 2013) and are the most frequent drug target of approved therapeutics, with around 35% of all drugs currently on the market acting on GPCRs (Sriram and Insel, 2018).

The canonical understanding of GPCR activation and signal transduction progresses in a linear fashion beginning with the activation of a transmembrane receptor by an extracellular ligand. The signal is then relayed to the intracellular environment via a conformational change of the receptor which allows the coupling of a heterotrimeric G-protein consisting of an α, a β, and a γ subunit to an intracellular domain of the active GPCR which facilitates nucleotide exchange and G-protein activation (Calebiro et al., 2021). In this classical progression, the Gα subunit, once dissociated from the Gβγ subunits, is the initiator of downstream signalling with different families of Gα proteins specifically mediating signalling via different signal transduction cascades. These include generation of the second messenger 3′,5′-cyclic adenosine monophosphate (cAMP), inositol triphosphate and diacylglycerol generation, Ca^2+^ release from intracellular stores, and activation of Rho family GTPases (Levitzki, 1988; Berridge, 2009; Siehler, 2009). Termination of the signal is then mediated by phosphorylation of the receptor by a G-protein coupled receptor kinase (GRK) which recruits β-arrestin (β-arr) to the receptor, terminating the signal, and facilitating receptor internalisation and degradation (Jean-Charles et al., 2017).

Over recent years, evidence has accumulated that the process is not this simple, and each step of this classical pathway has added layers of complexity. Receptor activation is more convoluted than a binary ‘on’ and ‘off’ switch with more recent evidence suggesting receptors have multiple active conformations which are stabilised by different ligands (Wingler and Lefkowitz, 2020). G-protein coupling and β-arr recruitment have also been topics of intense interest as the idea of ‘biased’ signalling has gained more evidence and its mechanisms have been investigated (Smith et al., 2018). Biased signalling refers to the ability of different ligands acting at the same receptor to preferentially activate G-protein-dependent or β-arr-dependent signal transduction. Similarly, second messenger signalling is not as homogenous as classical signal transduction models suggest in which receptor activation leads to either an increase or decrease in intracellular second messenger concentration homogeneously throughout the cell. Instead, second messenger levels are compartmentalised and regulated at the subcellular level, with distinct subcellular locations experiencing different intensity of the signal (McCormick and Baillie, 2014). Furthermore, GPCRs which were previously thought of as cell surface receptors have been identified at multiple intracellular locations including endosomes and mitochondria and these receptors initiate functionally relevant signal transduction independent of cell surface receptors [extensively reviewed in (Jong et al., 2018; Lohse et al., 2023; von Zastrow and Sorkin, 2021)]. Despite the large number of drugs on the market which target GPCRs, our understanding of how many of these drugs work is incomplete. Studying these more recently appreciated aspects of GPCR signalling may be key to understanding their mechanisms of action and developing new and better GPCR-targeted therapeutics.

Experiments using light-emitting biosensors have been used extensively to study all of these more recently described aspects of GPCR signalling in live cells. Here we review how fluorescent and bioluminescent biosensors can be used for studying GPCR signalling, giving illustrative examples of different sensor designs, comparing their advantages and disadvantages. We illustrate how using these tools has led to key findings and how these methodologies could impact the development of novel therapeutics in the future.

## 2 Sensors for receptor activation

The canonical two-state model of GPCR activation, in which a receptor exists in an equilibrium between inactive and active conformation and an agonist drives the equilibrium towards the active state fails to account for biased agonism and does not offer a full mechanistic explanation for partial and inverse agonism. Data suggest that GPCRs exist in multiple different active conformational states and that these conformations are stabilised by different ligands (Wingler and Lefkowitz, 2020). This model could provide a mechanistic explanation for biased ligands and could also underly partial and inverse agonism and offer a new explanation for ligand efficacy. One way of observing these multiple active conformations and measuring which conformation is stabilised by a given ligand is using intramolecular conformational biosensors based on resonance energy transfer. Additionally, nanobody-based conformational biosensors have been used to stabilise and study these differing receptor active-states. Both of these constructs are discussed in this section.

### 2.1 Intramolecular GPCR conformational sensors

Resonance energy transfer (RET) is a physico-chemical phenomenon in which an excited donor transfers energy to a fluorescent acceptor provided there is spectral overlap between them, and donor and acceptor are suitably close to each other (typically <10 nm). Generally, RET methods can be described as fluorescence resonance energy transfer (FRET) or bioluminescence resonance energy transfer (BRET). FRET uses a donor fluorophore which requires external excitation by direct illumination whilst in BRET, the energy donors are light-emitting enzymes such as luciferases.

Given that the efficiency of RET is highly sensitive to a change in the distance between donor and acceptor, by incorporating a RET pair into GPCRs, changes of FRET or BRET can be used to monitor conformational changes and thus receptor activation states in live cells upon ligand binding and receptor activation. Structural studies have shown that the greatest conformational change upon GPCR activation occurs within the cytoplasmic portion of the receptor. Rearrangement mediated especially by the outward displacement of transmembrane domain 6 (TM6) from TM3 and TM5 reveals the residues to which the C-terminus of a G-protein α-subunit binds (Kobilka, 2007; Weis and Kobilka, 2018; Hauser et al., 2021). For this reason, the intracellular loop 3 (ICL3) connecting TM5 and TM6 is typically the insertion site for one of the RET energy donor or acceptor in many intramolecular GPCR biosensors, while the other moiety is usually incorporated into the C-terminus.

**FIGURE 1:**
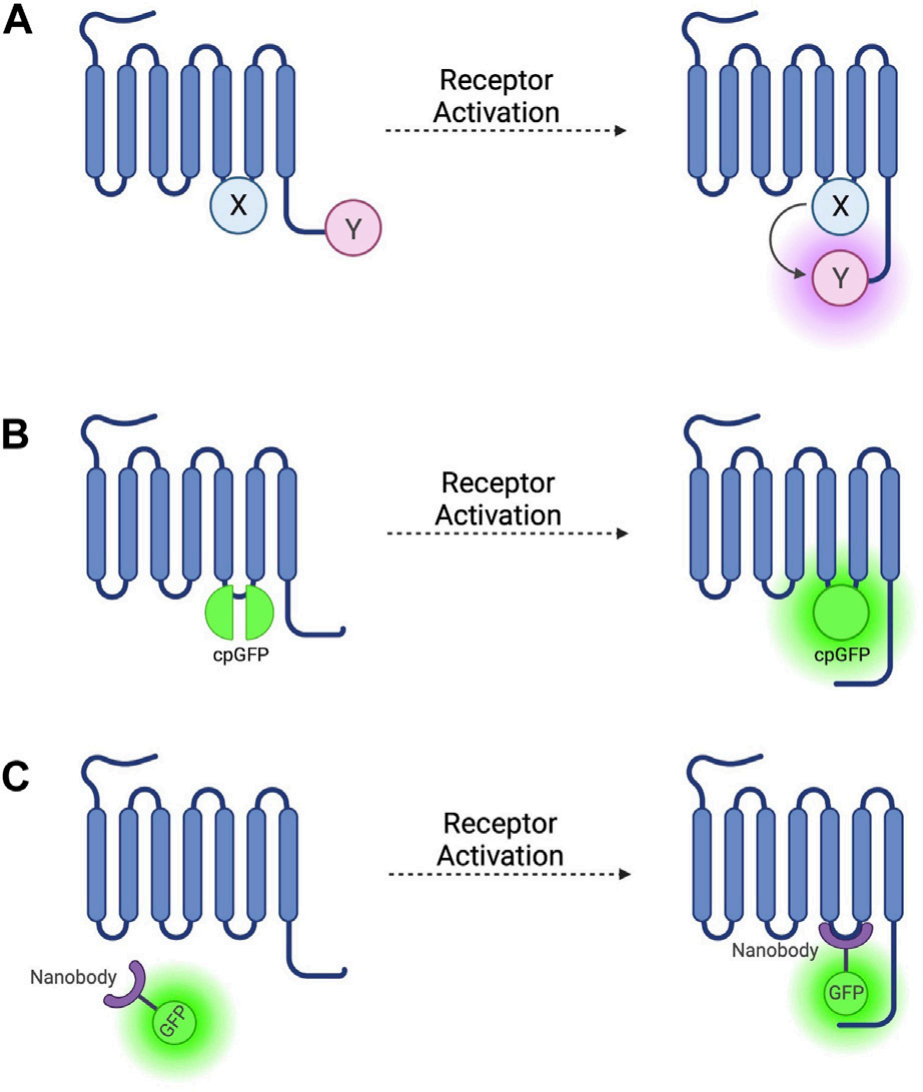
Schematic representations of sensors for GPCR activation **(A)** Generic intramolecular conformational RET sensor with energy donor X fused to intracellular loop 3 (ICL3) and energy acceptor Y fused to C-terminus. X and Y can be a CFP/YFP or CFP/FlAsH FRET pair or an RLuc/YFP or RLuc/FlAsH BRET pair **(B)** Intramolecular conformational sensor with circularly permuted GFP (cpGFP) fused to ICL3. **(C)** Fluorescent nanobody-based biosensor with GFP fused to camelid nanobody which binds to active-conformation ICL3.

The first GPCR FRET reporters were generated by conjugating a CFP donor to ICL3 and a YFP acceptor to the C-terminus (Figure 1A) of the α_2A_ adrenergic receptor (α_2A_-AR) and parathyroid hormone 1 receptor (PTH1R) (Vilardaga et al., 2003). Similar approaches have been used to generate FRET reporters for the β_1_ adrenergic receptor (β_1_-AR) (Rochais et al., 2007), β_2_ adrenergic receptor (β_2_-AR) (Reiner et al., 2010), and M1 muscarinic acetylcholine receptor (Jensen et al., 2009), amongst others. These constructs have the benefit of being completely genetically encoded, but the large size of the CFP fluorophore conjugated to ICL3 may disrupt conformational change via steric hinderance and can affect other intrinsic properties of the receptor, including ligand affinity and activation kinetics, and reduces the ability of the receptor to couple to G-proteins (Vilardaga et al., 2003).

To avoid these problems, YFP can be replaced as a FRET acceptor by fluorescein arsenical hairpin binder (FlAsH)—a fluorophore which binds the short six amino acid sequence CCPGCC–by incorporating this attachment sequence into a ICL3 (Figure 1A) (Hoffmann et al., 2005) and moving the CFP donor to the C-terminus. These reporters have been developed based on α_2A_-AR (Nikolaev et al., 2006b) and the adenosine 2A receptor (A_2A_R) (Fernandez-Duenas et al., 2014) and others (see Table 1). These constructs escape the issues associated with steric hinderance due to the fluorophore size being around 40 times smaller than GFP and its variants, meaning that the pharmacological properties of the receptor are conserved. However, CFP/FlAsH FRET pairs have the disadvantage of not being fully genetically encodable and requiring several complex washing steps in the labelling protocol (Hoffmann et al., 2010).

**TABLE 1:** Sensors discussed in Section 2 for detection of GPCR activation, ligand binding, and conformational change.

GPCR signalling step	GPCR target(s)	Detection method/mechanism	References(s)
GPCR conformational change	α_2A_-adrenergic receptor (α_2A_AR)	CFP/YFP FRET	Vilardaga et al. (2003)
		CFP/FlAsH FRET	Nikolaev et al. (2006b)
	Parathyroid hormone 1 receptor (PTH1R)	CFP/YFP FRET	Vilardaga et al. (2003)
	β_1_-adrenergic receptor (β_1_-AR)	Cerulean/YFP FRET	Rochais et al. (2007)
	β_2_-adrenergic receptor (β_2_-AR)	CFP/YFP FRET	Reiner et al. (2010)
	M_1_ and M_2_ muscarinic acetylcholine receptors	CFP/YFP FRET	Jensen et al. (2009), Maier-Peuschel et al. (2010)
		CFP/FlAsH FRET	
	Adenosine 2A receptor (A_2A_R)	CFP/YFP FRET	Hoffmann et al. (2005), Fernandez-Duenas et al. (2014)
		CFP/FlAsH FRET	
	Angiotensin II receptor 1 (AT_1_R)	RLucII/FlAsH BRET	Devost et al. (2017)
	Prostaglandin F receptor (FP)	RLuc/FlAsH BRET	Sleno et al. (2016)
	α_2A_-adrenergic receptor (α_2A_AR); β_2_-adrenergic receptor (β_2_-AR); parathyroid hormone 1 receptor (PTH_1_R)	NLuc/Halo BRET	Schihada et al. (2018)
	β_2_-adrenergic receptor (β_2_-AR), β_1_-adrenergic receptor (β_1_-AR)	Nanobody80-GFP (Nb80-GFP)	Irannejad et al. (2013)
	μ-opioid receptor (MOR), δ-opioid receptor (DOR)	Nb39-EGFP	Stoeber et al. (2018)
Ligand binding	Dopamine D_1_ receptor (DRD1), D_2_ receptor (DRD2), D_4_ receptor (DRD4)	Circularly permuted GFP (cpGFP) reconstitution	Patriarchi et al. (2018)
	Dopamine D_2_ receptor (DRD2)	Circularly permuted EGFP (cpEGFP) reconstitution	Sun et al. (2018)
	α_2A_-adrenergic receptor (α_2A_AR)	cpEGFP reconstitution	Feng et al. (2019)
	M_3_ muscarinic acetylcholine receptor	cpEGFP reconstitution	Jing et al. (2020)

Some FRET pair combinations suffer from low signal-to-noise ratios and samples can be bleached by the direct photoactivation required. BRET circumvents these problems but sacrifices the large dynamic range seen in FRET changes so smaller conformational changes may be missed. GPCR-based BRET biosensors have been developed by pairing either a YFP acceptor or a FlAsH acceptor with Renilla luciferase (RLuc) as a donor. Earlier constructs demonstrated small BRET changes but crucially, retained endogenous signalling properties (Sleno et al., 2016; Devost et al., 2017). The dynamic range of these biosensors has since been improved by changing the BRET donor/acceptor pairs used, using NanoLuc in place of RLuc and a variety of BRET acceptors (Schihada et al., 2018). In this way, BRET biosensor constructs have been developed based on the angiotensin II receptor (AT_1_R) (Szalai et al., 2012), Bradykinin 1 receptor (B_1_R) (Zhang et al., 2013) and the α_2_-AR, β_2_-AR and PTH1R (Schihada et al., 2018).

More recently, biosensors based on circularly permuted GFP variants (cpFPs) have been developed (Patriarchi et al., 2018; Sun et al., 2018) by incorporating the cpFP into receptors’ ICL3 (Figure 1B). These biosensors, rather than on RET, rely on a change in fluorescence intensity of a single cpFP upon conformational change of the receptor. The first such biosensors–dLight (Patriarchi et al., 2018) and GRAB_DA_ (Sun et al., 2018)—are dopamine receptor constructs used to detect endogenous dopamine release and extracellular dopamine concentration in live mice, zebrafish, and *drosophila*. Similar cpFP-GPCR constructs have been developed to detect other ligands, including noradrenaline (Feng et al., 2019), acetylcholine (Jing et al., 2020), and serotonin (Unger et al., 2020). These constructs have the advantage of being fully genetically encoded and, although very useful for ligand detection, reduced, or in some cases altogether abolished receptor-G-protein coupling as well as β-arr recruitment and receptor internalisation, make them less useful for studying downstream signalling.

Studies using intramolecular GPCR FRET and BRET conformational biosensors show that different ligands acting at the same receptor evoke RET changes with differing kinetics and magnitude with full agonists evoking the fastest response, followed by partial agonists, and then inverse agonists (Vilardaga et al., 2003; Nikolaev et al., 2006b; Fernandez-Duenas et al., 2014). Additionally, partial agonists induce smaller RET changes than full agonists, with inverse agonists causing RET changes opposite in direction to those of agonists. These observations are consistent with data which suggest the ability of individual GPCRs to adopt several different active conformational states (Wingler and Lefkowitz, 2020), suggesting that partial and inverse agonism could be mechanistically explained by ligands stabilising different conformations of the same receptor. The same could be true of biased agonism, with different active conformations preferentially coupling G-protein or β-arr mediated signalling. Although they indicate receptor activation, unlike RET sensors, tools based on circularly permuted fluorophores cannot be used to differentiate active conformations. Moreover, RET-based sensors show differences in FRET change in the presence of receptor allosteric modulators (Maier-Peuschel et al., 2010).

### 2.2 Nanobody-based conformational biosensors

Antibodies are typically heterotetramers of two heavy chains (V_H_) and two light chains (V_L_). By contrast, camelid species (e.g., dromedaries and llamas) produce functional heavy chain-only homodimers (V_HH_) (Hamers-Casterman et al., 1993). Nanobodies are made from the recombinant variable domains of V_HH_ antibodies and are around one-quarter the size of typical antibody fragments used for fluorescent labelling. In addition to being smaller in size, nanobodies have a more flexible region which recognises and interacts with the antigen, allowing them to access and bind to epitopes hidden within cavities inaccessible by conventional antibodies (Manglik et al., 2017). This property has led to the use of nanobodies to selectively bind to and stabilise different conformational states of GPCRs.

The first such nanobody to be generated was nanobody 80 (Nb80), a β_2_-adrenergic receptor-specific antibody which acts as a Gs protein mimetic and binds to the receptor G-protein binding pocket, which is only accessible when the receptor is in an active conformation (Rasmussen et al., 2011). Nb80 was developed into the prototypical genetically encoded nanobody-based GPCR biosensor by fusing the nanobody to enhanced green-fluorescent protein (GFP) (Figure 1C) (Irannejad et al., 2013). This Nb80-GFP construct can be used to indicate the presence and location of active βARs.

Similar nanobody-based GPCR sensors have been developed which bind to other active-state receptors, including *µ* opioid receptors (Huang et al., 2015), δ opioid receptors (DOR) (Stoeber et al., 2018) and M_1_ muscarinic acetylcholine receptors (Kruse et al., 2013) (see Table 1). Interferometry experiments with the *µ* opioid receptor nanobody Nb39 indicate that the nanobody binding signal is related to agonist efficacy, with higher efficacy agonists leading to higher nanobody association rates (Livingston et al., 2018). Like RET-based sensors, nanobody-based sensors could be useful in assays to determine agonist efficacy at GPCRs.

Experiments using these nanobody-based sensors suggest that ligands differentially activate receptors at specific subcellular locations and fluorescent nanobody-based biosensors can be used to visualise the location of active receptors within a cell. In this vein, nanobody-based sensors have been used to demonstrate the presence of active β-adrenergic receptors in early endosomes (Irannejad et al., 2013) and the Golgi apparatus (Irannejad et al., 2017) and the relevance of receptor subcellular location to compartmentalisation of GPCR signalling (Willette et al., 2023). By combining a receptor targeting fluorescent nanobody such as Nb80-GFP with Nb37, a nanobody which recognises Gαs, active GPCRs can be tracked through their internalisation and the co-localisation or separation of the two sensors can be used to determine Gαs recruitment and receptor activity (Irannejad et al., 2013). Like many antibody-based techniques, the use of these fluorescent nanobodies requires the over-expression of the target receptor so receptor location may not always represent the physiological localisation at endogenous expression levels. Another limitation of expressing nanobody based sensors intracellularly by transfection is the difficulty to control expression levels of the nanobodies themselves which, if too high, may interfere with downstream signalling. This problem can be minimised by using an inducible expression system (De Groof et al., 2021).

## 3 Sensors for G-protein-dependent signal transduction

The subsequent step to ligand binding in GPCR activation is the recruitment of a heterotrimeric G-protein composed of one each of a Gα, Gβ, and Gγ subunit. Gβ and Gγ are an obligate heterodimer and act as a single functional Gβγ subunit. In its resting state, the Gα subunit is bound to both the Gβγ subunit and a molecule of GDP. The conformational changes discussed in Section 2 expose the intracellular G-protein binding domain of the GPCR which then binds to the Gα subunit. Upon recruitment to an active receptor, the GDP is released and replaced by GTP. This active GTP-bound form of Gα dissociates from both the receptor and the Gβγ subunit and each of the Gα and Gβγ subunits go on to interact with further downstream effectors including adenylate cyclase, phospholipase-C, inwardly rectifying potassium channels, calcium channels and phosphoinositide-3-kinase (PI3K) (Calebiro et al., 2021). There are still many gaps in our understanding of this process. For example, most GPCRs signal predominantly through one of the G_s_, G_i_, G_q_, or G_12/13_ pathways but many receptors seem to be able to couple to more than one subtype of G-protein. Additionally, multiple studies support G-protein-independent signal transduction but how G-protein-dependent and G-protein-independent signalling is balanced at the receptor is not well understood. Fluorescent and bioluminescent biosensors have been developed which have been used to interrogate several steps of this cascade, including G-protein recruitment, conformational change, and activation. All sensors discussed in this section are summarised in Table 2.

**TABLE 2:** Sensors discussed in Section 3 for detection of G-protein recruitment to GPCRs and G-protein dissociation and activation.

Sensor type	GPCR signalling step	Detection method/mechanism	References
Intermolecular G-protein sensor	G-protein heterotrimer dissociation	CFP/YFP FRET	Janetopoulos et al. (2001)
		RLuc/GFP BRET	Olsen et al. (2020)
		“TRUPATH”	
		NanoLuc/cpVenus BRET “G-CASE”	Schihada et al. (2021)
		NanoBiT complementation assay	Inoue et al. (2019)
	G-protein recruitment to GPCR	CFP/YFP FRET	Hein et al. (2005)
		RLuc/GFP BRET	Gales et al. (2005)
		RLuc/venus BRET with mini-G	Wan et al. (2018)
		NanoBiT complementation assay with full G-protein	Laschet et al. (2019)
		NanoBiT complementation assay with mini-G	Benkel et al. (2022)
	G-protein recruitment to membrane	RLuc/Venus bystander BRET	Martin and Lambert (2016)
		NanoLuc/Venus bystander BRET	Wan et al. (2018)
		RLuc/rGFP ebBRET	Wright et al. (2021)
		RLuc/rGFP ebBRET EMTA	Avet et al. (2022)
	Gβγ interaction with GRK	Cerulean/BiFC Venus FRET	Hollins et al. (2009)
		RLuc/BiFC Venus BRET	Fuchs et al. (2013), Masuho et al. (2015)
		NanoLuc/BiFC Venus BRET	Masuho et al. (2021)
		RLuc/GFP10 BRET	Karamitri et al. (2018)
Unimolecular G- protein sensor	G-protein recruitment to GPCR	mCerulean/mCitrine FRET “SPASM”	Sivaramakrishnan and Spudich (2011)
	G-protein activation (nucleotide exchange)	NanoLuc/YFP BRET “BERKY”	Maziarz et al. (2020)

### 3.1 G-protein intermolecular RET sensors

**FIGURE 2:**
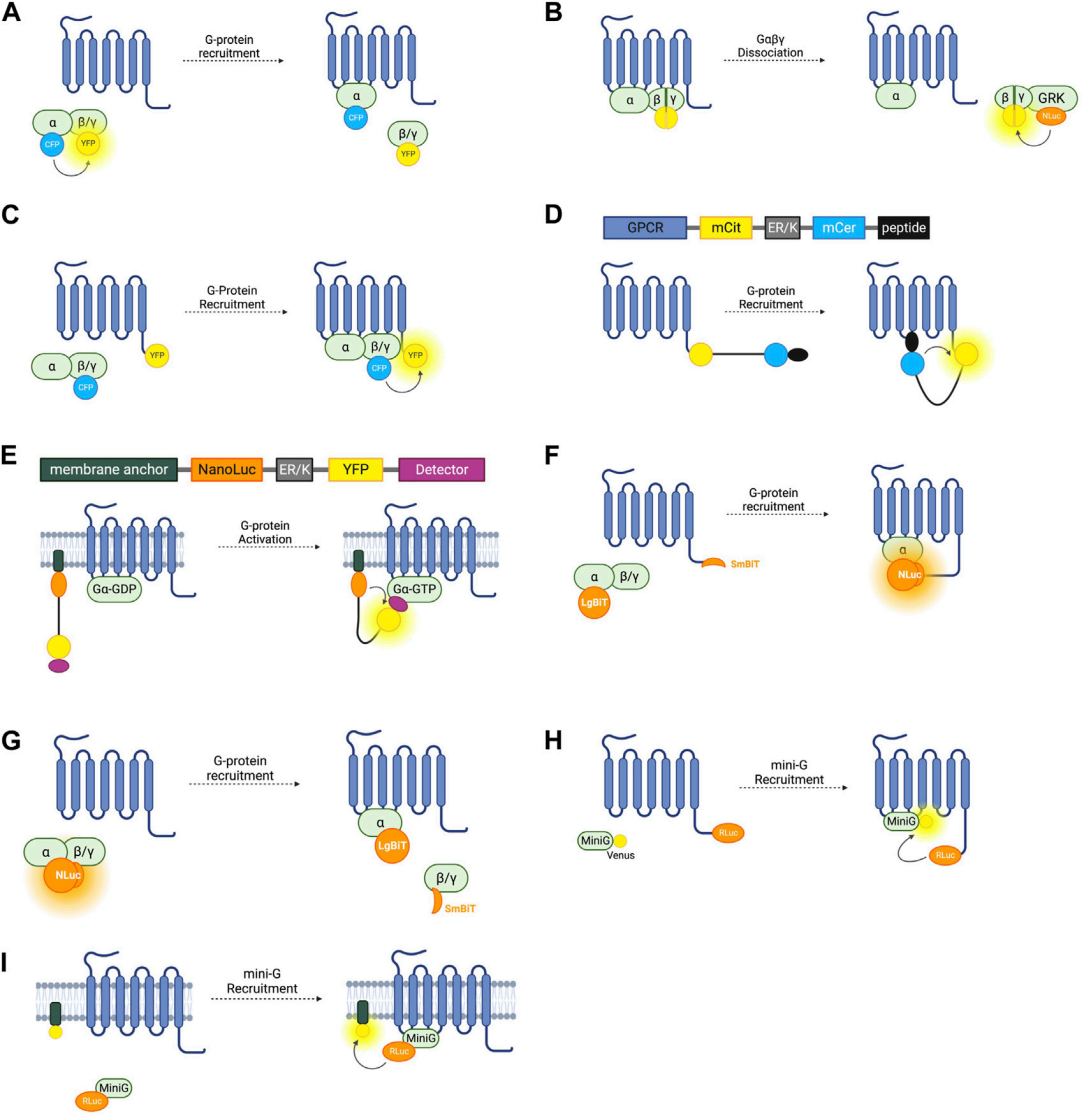
Schematic representations of sensors for GPCR/G-Protein coupling **(A)** Intermolecular FRET sensor for G-protein recruitment with CFP donor fused to Gα subunit and YFP acceptor fused to Gβγ subunit. **(B)** Intermolecular BRET sensor for Gβγ/GRK interaction with NanoLuc donor fused to GRK and Venus acceptor formed by bimolecular fluorescence complementation (BiFC) between Gβ and Gγ subunits. **(C)** Intermolecular FRET sensor for G-protein recruitment with CFP donor bound to Gβγ subunit and YFP acceptor bound to receptor C-terminus. **(D)** Systematic protein affinity strength modulation (SPASM) sensor consisting, from N- to C-terminus, of a GPCR, mCit FRET acceptor, ER/K linker, mCer FRET donor, and peptide which binds ICL3 upon receptor activation. **(E)** BRET sensor with ER/K linker and YFP (BERKY) consisting, from N- to C-terminus, of membrane anchor, NanoLuc BRET donor, ER/K linker, YFP BRET acceptor and detector peptide which selectively binds the Gα subunit in its GTP bound state. **(F)** NanoBiT sensor for G-protein recruitment with the large NanoLuc fragment LgBiT fused to the Gα subunit and the small NanoLuc fragment SmBiT fused to the receptor C-terminus. **(G)** NanoBiT sensor for G-protein dissociation with LgBiT fused to the Gα subunit and SmBiT fused to the Gβγ heterodimer. **(H)** MiniG BRET sensor with RLuc BRET donor fused to receptor C-terminus and Venus BRET acceptor fused to MiniG. **(I)** MiniG bystander BRET sensor with RLuc donor fused to the MiniG protein and BRET acceptor anchored to the membrane by a membrane localisation sequence.

The first intermolecular G-protein based FRET sensors used a CFP donor and YFP acceptor fused to an internal loop of the Gα- and the N-terminus of the Gβ- G-protein subunit respectively (Figure 2A) (Janetopoulos et al., 2001). Given the importance of the N- and C-termini of the Gα protein for its interaction with receptors, downstream effectors, and Gβγ heterodimers, the CFP is inserted into the first loop between αA and αB, allowing the Gα protein to remain functional. These constructs are fully genetically encoded and provide a useful system for studying the G-protein cycle and G-protein activation kinetics. On G-protein activation these sensors typically show the expected decrease in FRET caused by Gα and Gβγ dissociation. However, experiments using these sensors with Gαi-coupled receptors have demonstrated an occasional increase in FRET on receptor activation, indicating that the α- and βγ-subunits do not always dissociate fully, but rather downstream G-protein signalling may be facilitated by rearrangement of the heterotrimer rather than disassociation (Bunemann et al., 2003).

Free Gβγ sensors have also been used to study G-protein heterotrimer dissociation. The FRET or BRET acceptor in this case is fluorescent protein Venus of which split complementary fragments are fused to Gβ and Gγ. Co-expression of these constructs results in Gβγ heterodimers labelled with Venus through Bimolecular Fluorescence Complementation (BiFC) (Hollins et al., 2009). These constructs can be co-expressed with a FRET or BRET donor fused to a C-terminal fragment of a GPCR-kinase (GRK) which binds to free Gβγ following G-protein heterotrimer dissociation, resulting in an increase in BRET or FRET (Figure 2B). Both RLuc8 and cerulean donors have been used in these constructs for BRET or FRET respectively (Hollins et al., 2009; Fuchs et al., 2013) and a GFP10 acceptor has been used in place of Venus (Karamitri et al., 2018). By overexpressing each Gα-subunit and co-expressing the Gβγ, and GRK constructs, the Gα-specificity of a receptor can be interrogated (Karamitri et al., 2018). This system is capable of monitoring activation of each of the four major Gα families (Masuho et al., 2015). One advantage of these constructs is that they allow assessment of endogenous GPCR-Gα coupling as neither the receptor, nor the Gα subunit are tagged with a fluorescent or bioluminescent protein, meaning there is minimal interference with Gα-recruitment. One drawback is that this requires the co-transfection of at least four separate constructs. Interestingly, the Gβγ BiFC heterodimers have also been used independently of this BRET or FRET setup to study Gβγ signalling, including determining the propensity of specific Gβ and Gγ proteins to form heterodimers and the differing signalling properties of these (Masuho et al., 2021).

G-protein recruitment to active receptors has also been studied by fusing one fluorophore to the receptor itself, and another to a G-protein subunit (Figure 2C). An increase in FRET ratio in this case indicates G-protein recruitment. The first such constructs were developed by fusing a CFP donor to the γ-subunit N-terminus, and a YFP acceptor to the C-terminus of the α2A-adrenergic receptor (Hein et al., 2005). Like the intramolecular RET sensors discussed in Section 2.1, the energy donor can be replaced with a luciferase and BRET signal used instead of FRET (Gales et al., 2005). Using BRET has the advantage of not requiring external excitation so this tool can be used in a 96-well plate and a standard plate reader, meaning that it is more suitable for higher-throughput screening assays, although it does not have the same sensitivity to small changes in fluorophore distance as the FRET sensors. Both of these approaches rely on the overexpression of G-proteins in living cells which may artificially affect the rate of G-protein recruitment.

Due to differences in insertion sites for FRET/BRET donors and acceptors and incomplete characterisation of newly generated constructs, often experiments using this approach are not comparable. To circumvent this problem, the TRansdUcer PATHways (TRUPATH) collection was developed (Olsen et al., 2020). TRUPATH is an experimentally optimised open-source suite of Gαβγ BRET sensors using an RLuc8 donor and GFP2 acceptor. A number of insertion sites for the luciferase donor within either the αA-αB or αB-αC loop regions were tested for each of the 16 human Gα-protein subunits. For each, the construct which exhibited the largest dynamic range was selected. For two of the Gα-proteins, no constructs trialled were functional. In a stepwise process, 12 Gγ proteins with an N-terminal GFP, and 4 Gβ proteins were tested with each Gα construct and the combination which resulted in the greatest dynamic range upon activation of a cognate receptor was selected for the TRUPATH suite. Available through Addgene (Addgene kit #1000000163), the suite covers 14 different Gα subunits paired with 2 Gβ subunits, and 4 Gγ subunits which can be used in combination to form biosensors covering 14 different G-protein pathways. This strategy has been further developed for use in a 384-well plate format, facilitating higher throughput for agonist and antagonist screening (DiBerto et al., 2022).

Despite the improved sensitivity of the sensors in the TRUPATH toolkit resulting from this extensive in-cell optimisation, use of these sensors requires co-transfection with three separate plasmids, each encoding one of the α, β, and γ G-protein subunits. This leads to difficulties ensuring equal expression of each of the subunits which in turn causes variation in BRET signal between cells. G-CASE sensors (G-protein tri-cistronic activity sensors) were designed with this problem in mind and encode all three G-protein subunits in one plasmid (Schihada et al., 2021). Like TRUPATH, G-CASE is a suite of BRET sensors composed of NanoLuc tagged Gα-subunits, and circularly permuted Venus (cpVenus) tagged Gγ-subunits. The insertion sites for the NanoLuc donor are taken either from TRUPATH, or previously published G-protein FRET sensors, while the cpVenus acceptor is fused to the Gγ N-terminus. To facilitate expression from a single plasmid, two strategies used by viruses to express multiple proteins from one transcript were taken advantage of. The Gβ and Gγ are linked by a viral 18 amino acid T2A sequence which is cleaved co-translationally, separating the two subunits (Szymczak and Vignali, 2005). To allow co-expression, the Gα-subunit is encoded downstream of an internal ribosome entry site (IRES) which facilitates ribosome binding and translation of the Gβγ sequence (Szymczak and Vignali, 2005). This approach was applied to a spectrum of Gα, Gβ, and Gγ proteins, resulting in 8 plasmids for 8 distinct Gα-signalling pathways.

### 3.2 G-protein unimolecular RET sensors (SPASM and BERKY)

To overcome the difficulty of ensuring equal expression of individual donor and acceptor fluorophores, a chimeric approach has been developed termed Systematic Protein Affinity Strength Modulation or SPASM (Sivaramakrishnan and Spudich, 2011). SPASM utilises a unique ER/K alpha helical linker protein composed of an alternating sequence of approximately four glutamic acid (E) residues, followed by four arginine (R), or lysine (K) residues (Sivaramakrishnan and Spudich, 2011). This linker can be used to join two interacting proteins which are each fused to a donor or acceptor fluorophore. The flexible linker allows the protein-protein interactions required for RET, and changes in the interaction are measured by recording changes in FRET ratio.

This system has been applied to GPCR-G-protein interaction with the first GPCR SPASM sensors developed for the β2-adrenergic receptor and opsin (Malik et al., 2013). From N- to C-terminus, the sensors are composed of the GPCR of interest, mCitrine (which acts as the FRET acceptor), ER/K linker, mCerulean (FRET donor), and a 27 amino acid polypeptide fragment of the relevant Gα-subunit (Figure 2D). Upon activation of the receptor, the Gα-protein fragment binds, resulting in an increase in FRET. By varying this protein fragment, sensors for Gα_s_, Gα_i_, and Gα_q_ were developed, as well as an additional construct using a protein fragment with high affinity for activated rhodopsin. Using only a C-terminal fragment of the Gα protein and not the full subunit may change the selectivity of the receptor for the G-protein and so may not be representative of their natural interaction. For this reason, GPCR SPASM sensors have been further developed by incorporating the full Gα-subunit (Malik et al., 2017). This construct retains the ability to bind the Gβγ subunit and preserves downstream signalling.

The study of G-protein coupling and activation dynamics using SPASM sensors suggested that a single ligand-binding event at a receptor may couple to the activation of several G-protein heterotrimers with the first interaction between a G-protein and receptor ‘priming’ the receptor and leaving it in an altered conformational state for up to one and a half minutes, during which time G-protein interaction is enhanced (Gupte et al., 2019). Another aspect of G-protein signalling that has been studied using SPASM sensors is G-protein selectivity. Many receptors are classified as only Gα_s_, Gα_i_, Gα_q/11_, or Gα_12/13_ coupled and, in many cases, this is justified as they are well characterised as signalling predominantly via that pathway–for example, the β_1_-AR coupling to Gα_s_. However, many receptors exhibit promiscuous G-protein coupling, showing some degree of interaction with several different G-proteins. The SPASM system has been shown to work for all of these G-protein classes and when an ER/K linker is used to join a non-cognate GPCR-G-protein pair, this does not artificially induce coupling (Mackenzie et al., 2019) allowing SPASM sensors to be used to assay the selectivity of GPCRs for G-protein classes. Modifying the approach by using a NanoLuc and mCit BRET pair further allows this system to be applicable as high throughput screening in a standard plate reader (Mackenzie et al., 2019).

G-protein recruitment to an active GPCR, although a good proxy, is not a direct measure of G-protein activation. A sensor which can indicate Gα-GTP formation would give the most direct readout of nucleotide exchange–the principal step of G-protein activation. The BRET Sensor with ER/K linker and YFP (BERKY) does just that. BERKY sensors are unimolecular constructs composed of an N-terminally membrane anchored NanoLuc BRET donor joined by an ER/K linker to a YFP BRET acceptor fused to a detector protein (Figure 2E) (Maziarz et al., 2020). The detector protein is able to reversibly and selectively bind to Gα-GTP without impeding nucleotide exchange or downstream signal transduction. In its resting state, the ER/K linker separates the NLuc donor and YFP acceptor sufficiently for minimal detectable basal BRET. Upon Gα-GTP binding to an active GPCR, the detector protein binds to the Gα-GTP, bending the ER/K linker and bringing the NLuc and YFP closer together, leading to increased BRET. By varying the detector protein, BERKY sensors have been developed for Gαi-GTP, Gαq-GTP, and Gα12-GTP (Maziarz et al., 2020), but there is currently no sensor for Gαs-GTP. In addition to active Gα subunits, further variation of the detector module has allowed the development of these constructs as biosensors for free Gβγ heterodimers and active Rho-GTPases (Maziarz et al., 2020). The former uses the C-terminal domain of GPCR kinase 3 (GRK3) as the Gβγ binding protein. GRK3 is recruited to the membrane by Gβγ after GPCR activation where it phosphorylates active receptors, recruiting beta-arrestin and terminating their signal (Daaka et al., 1997). This is discussed further in section 4.0. BERKY sensors are relatively new and have considerable potential for studying G-protein coupling and activation. The ability to detect Gα-GTP is unique to these sensors, and they are also the only sensors outlined in this section which do not require the overexpression or structural modification of a GPCR or G-protein, which makes them ideal for *in vivo* experiments or for studying downstream signalling.

### 3.3 NanoBiT-based sensors

NanoLuc (NLuc), is a bioluminescent reporter engineered using a luciferase subunit from a luminous deep-sea shrimp (Hall et al., 2012). Its small size means that labelled endogenous proteins incur less steric interference than is seen with larger fluorescent proteins or the bioluminescent Renilla luciferase (RLuc) (Hall et al., 2012). NLuc has been used to develop a split complementary bioluminescent reporter called NanoLuc Binary Technology (NanoBiT). The small NanoBit partner (SmBiT, 11 amino acids) and the large NanoBiT partner (LgBiT, 17.6 kDa) can be fused to separate proteins and, upon protein-protein interaction, NLuc is reconstituted resulting in luminescence (Dixon et al., 2016). This system has been used to investigate GPCR-G-protein interactions by fusing the SmBiT to the C-terminus of the receptor of interest, and the LgBiT to the G-protein α-subunit (Figure 2F) (Laschet et al., 2019). Proof of concept experiments testing recruitment to the β_2_-AR, D_2_-dopamine receptor, histamine receptor H_1_, and thromboxane A_2_ receptor show that this system is applicable to study Gα_s_, Gα_i_, Gα_q/11_, and Gα_12/13_, respectively, and can be used as high-throughput screening (Laschet et al., 2019). As well as having improved sensitivity over FRET and BRET approaches, this system only requires the expression of the Gα-subunit, not the full heterotrimeric G-protein complex but still relies on roughly equal stoichiometric expression of the SmBiT and LgBiT-fused proteins. One concern with using a complementation assay based on a split sensor is that it may not report the true dynamics of protein interactions due to the innate affinity of the two fragments of the split reporter, which may disturb the rate of GPCR-G-protein association or dissociation.

NanoBiT has additionally been used to develop sensors similar to the Gαβγ dissociation sensors relying on FRET and BRET discussed earlier. By inserting the LgBiT into the αA-αB internal loop region in a Gα subunit and fusing the SmBiT to the N-terminus of either a Gβ subunit, the dissociation of Gα and Gβγ is detectable by a decrease in bioluminescence intensity (Figure 2G) (Inoue et al., 2019). These sensors were used as part of a screening pipeline to determine G-protein coupling selectivity of a range of GPCRs and G-protein signal bias of ligands acting at these receptors. These experiments provided further evidence that many GPCRs are capable of coupling to multiple types of Gα (Inoue et al., 2019). A high throughput assay to assess G-protein selectivity would be useful in screening drug candidates and this system has potential to do just that but difficulties ensuring equal levels of expression of the Gα-LgBiT and Gβ-SmBiT constructs and untagged Gγ posed by co-transfection of three plasmids should be considered when using this approach.

### 3.4 Mini-G protein sensors

A Mini-G protein is the minimal Gα-protein fragment which still binds to its cognate GPCR and was first developed to facilitate crystallisation of GPCRs in their active conformation (Carpenter and Tate, 2016). The binding between a GPCR and a mini-G protein is irreversible as they are unable to facilitate nucleotide exchange so cannot dissociate from the active G-protein once bound. As well as enabling GPCR purification and structural characterisation, fluorescent mini-G chimeras have been developed to study GPCR-G-protein coupling. The first mini-G generated was mini-G_s_ (Carpenter and Tate, 2016), and since then, mini-G_i_, mini-G_q_, and mini-G_12_ have been developed (Nehme et al., 2017).

A BRET spectroscopy assay using mini-G proteins in which RLuc is fused to a GPCR and the GFP variant Venus is fused to the mini-G-protein (Figure 2H) can indicate G-protein recruitment (Wan et al., 2018). Mini-G_s_ and mini-G_12_ both express well in mammalian cells whilst mini-G_i_ and mini-G_q_ form intracellular aggregates, so mini-G_s/i_ (a chimera of mini-G_s_ and mini-G_i_) and mini-G_s/q_ (a chimera of mini-G_s_ and mini-G_q_) can be used instead (Carpenter and Tate, 2016; Wan et al., 2018). Given the cytosolic localisation of the fluorescent mini-G chimeras, the signal to noise ratio of this BRET assay is much higher than in the case of assays which rely on membrane anchored constructs and has been used to accurately profile G-protein coupling to an assortment of receptors, including the α_2A_- and β_2_-adrenergic receptors, A_1_ and A_2A_ adenosine receptors, D_1_, D_2_, and D_5_ dopamine receptors, and M_3_ and M_4_ muscarinic acetylcholine receptors (Carpenter and Tate, 2016). In addition to a BRET pair, the mini-G assay can be adapted to use the NanoLuc complementation assay NanoBiT (Section 3.3) by fusing LgBiT to the mini-G protein and SmBiT to the receptor of interest (Benkel et al., 2022). Although confocal microscopy and BRET spectroscopy have demonstrated mini-G recruitment to GPCRs at internal membranes such as the Golgi and endosomes (Wan et al., 2018), mini-G protein binding to a GPCR have been reported to abolish β-arr recruitment and subsequent receptor internalisation (Manchanda et al., 2021), which, if corroborated, would make them unsuitable to study internalised receptor signalling or biased agonism.

### 3.5 Bystander BRET-based G-protein sensors

The BRET-based sensors discussed so far in this section rely on a BRET signal generated by two interacting proteins each fused to a BRET donor or acceptor. This is referred to as ‘specific’ BRET. BRET can also occur when the two tagged proteins do not directly interact but are localised within the same compartment and thus within the 10 nm proximity required for energy transfer to occur. This is referred to as ‘nonspecific’ or ‘bystander’ BRET. By inserting an RLuc8 BRET donor into a loop in Gα_s_ and co-expressing this with membrane compartment markers tagged with a Venus BRET acceptor, bystander BRET has been used to explore the intracellular distribution of Gα_s_ subunits (Figure 2I) (Martin and Lambert, 2016). The BRET signal in cells co-expressing the luminescent Gα_s_ construct and Venus-K-Ras which localises to the plasma membrane was significantly larger than when co-expressed with Venus-PTP1b or Venus-giantin which localise to the endoplasmic reticulum (ER) and Golgi apparatus respectively. All three of these experimental setups resulted in BRET, demonstrating the presence of Gαs at the ER and Golgi, as well as in early, late, and recycling endosomes by using other Venus constructs.

Enhanced bystander BRET (ebBRET) was developed for an improved BRET signal (Namkung et al., 2016). Unlike most BRET-based assays which make use of a donor/acceptor pair from different species–usually RLuc from *Renilla reniformis* and a GFP variant from *Aequorea victoria*, ebBRET takes advantage of the naturally occurring pair from *R. reniformis* (RLuc and rGFP) which more efficiently transfer energy. ebBRET has been used to develop a Gα_q_ translocation assay to track the movement of Gα_q_ through the cell after activation of a cognate receptor (Wright et al., 2021). This assay made use of the mini-G_s/q_ chimera (mGsq) tagged with RLuc8, rGFP-CAAX which localises to the plasma membrane, and rGFP-FYVE which localises to early endosomes. Stimulation of Gα_q_-coupled angiotensin II receptor AT_1_R resulted in a short-term increase in BRET both at the plasma membrane and in early endosomes whilst with prolonged angiotensin II exposure, there was a decrease in BRET signal in rGFP-CAAX expressing cells and an increase in BRET in cells expressing rGFP-FYVE, representative of redistribution of active Gα_q_ from the plasma membrane to the early endosomal compartment. The ability of these constructs to indicate active GPCRs in different subcellular compartments, makes them useful for interrogating the presence of active GPCRs at internal membranes. Using a similar approach with a NanoLuc-fused mGsi and Venus targeted to different internal membranes, the presence of active A_1_-adensoine receptors at the Golgi apparatus has been demonstrated (Wan et al., 2018).

Instead of tagging the Gα-subunit with the luciferase, RLuc can be conjugated to a G-protein effector. This is the theory behind the ebBRET-based G-protein effector membrane translocation assay (EMTA) (Avet et al., 2022). In this assay, RLuc is fused to the C-terminus of p63-RhoGEF, Rap1GAP and PDZ-RhoGEF which selectively interact with activated Gα_q_, Gα_i_, and Gα_12/13_ respectively and the rGFP BRET acceptor is fused to a membrane localisation sequence. An advantage of these bystander BRET-based approaches is that they use the endogenous untagged receptor so do not have the limitations associated with experiments relying on receptor over-expression. However, it should be considered that when using bystander BRET, an increased BRET signal is nonspecific and so off-target activation of a different receptor may also recruit the G-protein to the membrane, resulting in a BRET signal so another assay should be used to confirm results where available.

## 4 β-arrestin-dependent signalling

After G-protein recruitment and activation, signal transduction must be terminated, and this is achieved via a two-step process. Active GPCRs are first phosphorylated by GPCR kinases (GRKs) and, subsequently, β-arr binds to the phosphorylated receptor (Jean-Charles et al., 2017). Through both steric hinderance of G-protein coupling and by acting as a scaffold for other proteins, β-arr recruitment results in the termination of the G-protein dependent signal. Amongst other effectors, arrestins scaffold cyclic nucleotide phosphodiesterases (PDEs) and diacylglycerol kinases (DGKs) which degrade the second messengers–cAMP and DAG–downstream of Gα_s_ and Gα_q_ coupled receptors, respectively. The consequent attenuation of protein kinase A (PKA) and protein kinase C (PKC)-mediated phosphorylation of GPCRs can additionally oppose further G-protein signalling (Jean-Charles et al., 2017). Recruitment of arrestin to receptors also induces receptor endocytosis and internal trafficking, initially thought to be solely for the purposes of GPCR degradation and desensitisation.

However, a body of work has accumulated demonstrating that arrestins are themselves important transducers of GPCR signals via G-protein alternative pathways (Gurevich and Gurevich, 2019). Arrestins have been shown to mediate signalling via the mitogen activated protein kinases (MAP kinases) ERK 1/2, JNK3, and p38; and via PI3K and protein kinase B (PKB), also known as Akt. Additionally, β-arr signalling has been demonstrated to mediate cytoskeletal rearrangement and transcriptional regulation (DeWire et al., 2007). β-arr recruitment is of therapeutic interest for a myriad of reasons. Firstly, β-arr-mediated GPCR desensitisation is linked to diminishing drug effects with repeated dosing (Ahn et al., 2003). More recently, β-arr-ergic signalling has been the focus of studies into biased agonism. This describes the phenomenon that whilst ‘balanced’ agonists equally induce G-protein-dependent and β-arr-dependent signalling, some so-called ‘biased’ agonists specifically amplify downstream pathways via one of these two arms over the other (Smith et al., 2018). This has attracted great research interest with several ligands which have β-arr biased agonism having been identified to date (Hodavance et al., 2016). Given many relatively recent advances in understanding biased agonism, little is known about the bias of many GPCR-targeted drugs currently on the market. There are several assays available relying on fluorescent and bioluminescent biosensors which can further our understanding in this area (see Table 3), and aid future drug development with a focus on biased agonism.

**TABLE 3:** Sensors discussed in Section 4 for detection of beta-arrestin (β-arr) and GPCR kinase (GRK) recruitment to GPCRs and β-arr conformational change.

Sensor type	GPCR signalling step	Detection method/mechanism	References
Intermolecular beta-arrestin sensor	β-arr recruitment to GPCR	RLuc/YFP BRET	Angers et al. (2000)
		RLuc/EYFP BRET	Kroeger et al. (2001)
		ECFP/EYFP FRET	Kraft et al. (2001)
		FlAsH/ReAsH FRET	Zurn et al. (2010)
		NanoBiT complementation assay	Dixon et al. (2016), He et al. (2018)
	β-arr recruitment to membrane	RLuc/citrine BRET	Clayton et al. (2014)
		RLuc/rGFP BRET	Namkung et al. (2016)
		Split NanoLuc complementation assay	Hauge Pedersen et al. (2021)
Intermolecular GRK sensor	GRK recruitment to GPCR	RLuc/YFP BRET	Hasbi et al. (2004)
		RLuc/GFP BRET	Jorgensen et al. (2008)
Intramolecular beta-arrestin RET sensor	β-arr conformational change	RLuc/YFP BRET	Charest et al. (2005)
		RLuc/FlAsH BRET	Lee et al. (2016)
		NanoLuc/CyOFP1 BRET	Oishi et al. (2020)
		CFP/FlAsH FRET	Nuber et al. (2016)

### 4.1 Intermolecular sensors for β-arrestin and GRK recruitment

**FIGURE 3:**
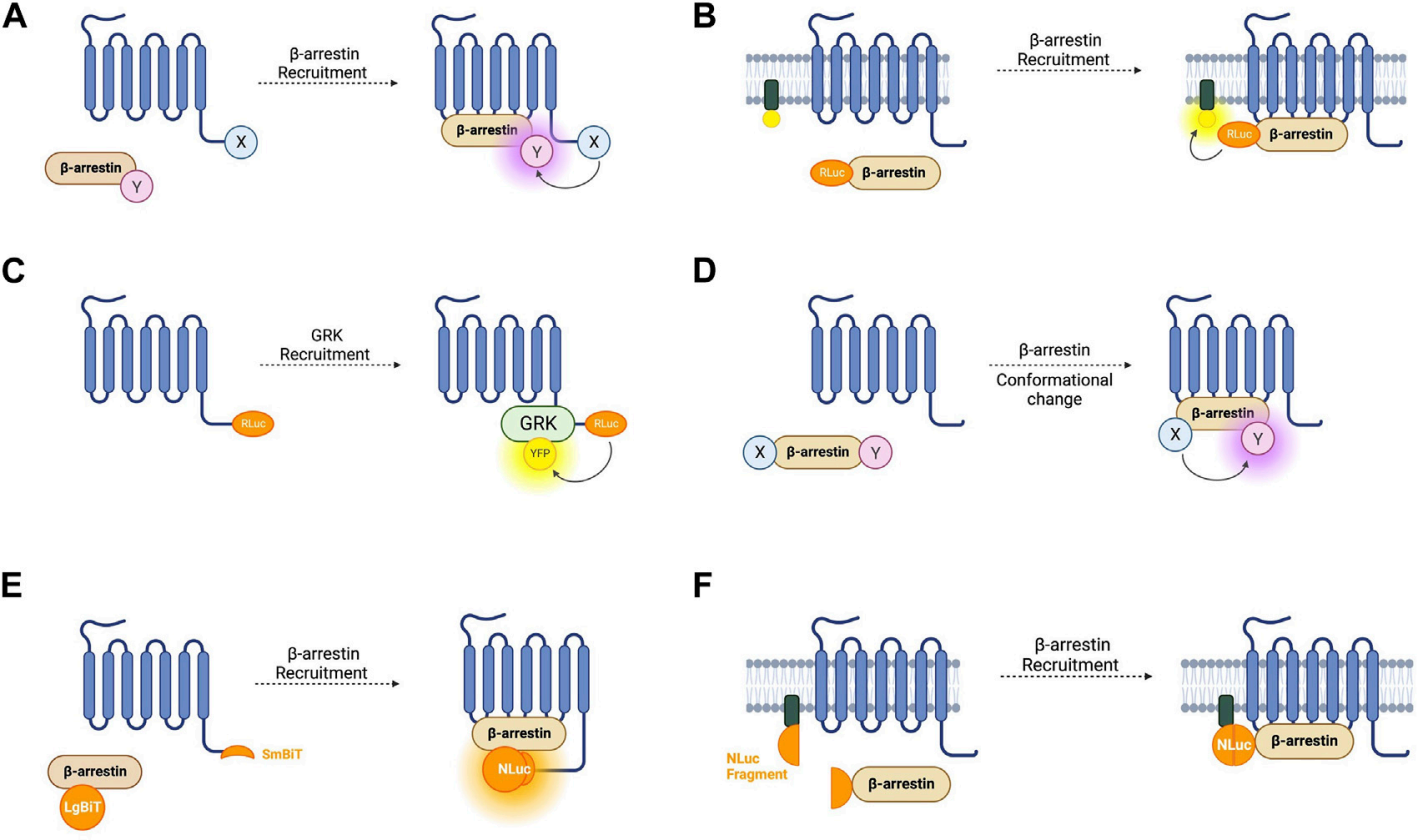
Schematic representations of sensors for GPCR/beta-arrestin (β-arr) and GPCR kinase (GRK) interaction **(A)** Generic intermolecular RET sensor for β-arr recruitment with energy donor X fused to GPCR C-terminus and energy acceptor Y bound to β-arr. X and Y can be and RLuc/YFP BRET pair, a CFP/YFP FRET pair, or a FlAsH/ReAsH FRET pair **(B)** Bystander BRET sensor for β-arr recruitment with RLuc donor fused to β-arr and BRET acceptor anchored to the membrane by a membrane localisation sequence. **(C)** BRET sensor for GRK recruitment with RLuc donor fused to GPCR C-terminus and BRET acceptor fused to GRK. **(D)** Generic intramolecular RET sensor for β-arr conformational change with energy donor X fused to β-arr N-terminus and energy acceptor Y fused to β-arr C-terminus. X and Y can be an RLuc/YFP BRET pair, an RLuc/FlAsH BRET pair, NanoLuc/cyOFP1 BRET pair, or a CFP/FlAsH FRET pair. **(E)** NanoBiT sensor for β-arr recruitment with large NanoLuc fragment LgBiT fused to β-arr and small NanoLuc fragment SmBiT fused to GPCR C-terminus. **(F)** Bystander split NanoLuc sensor for β-arr recruitment with one NanoLuc fragment fused to β-arr and the other complementary fragment anchored to the membrane by a membrane localisation sequence.

The first cell-based assay for studying GPCR-β-arr interaction was a BRET-based intermolecular assay in which the β_2_-AR with a C-terminally fused RLuc donor and β-arr with a C-terminally fused YFP acceptor were coexpressed (Figure 3A) (Angers et al., 2000). As expected, this biosensor demonstrated a dose-dependent agonist-induced BRET increase representative of β-arr recruitment to an active, phosphorylated receptor. Using a similar approach with YFP replaced by enhanced YFP (EYFP) as the energy acceptor for improved brightness, this system was shown to be generalisable as a high throughput assay used in 96-well plates (Kroeger et al., 2001). By using EYFP tagged β-arr and either complementary RLuc tagged thyrotrophin-releasing hormone receptors (THRH) or gonadotrophin-releasing hormone receptors (GnRHR), this assay showed proof of principle that it can differentiate receptors which do bind β-arr upon agonist stimulation (such as TRHR) and those which do not (including GnRHR) (Kroeger et al., 2001). Additionally, by expressing either β-arr1/GFP or β-arr2/GFP, the same group demonstrated that TRHR1 recruits both β-arr1 and β-arr2 equally whilst TRHR2 recruits only β-arr2 and not β-arr1 (Hanyaloglu et al., 2002). Furthermore, it has been shown that an individual receptor can selectively recruit either β-arr1 or β-arr2 when stimulated by different agonists (Hoffmann et al., 2008).

As discussed earlier, FRET sensors usually produce stronger signals and have higher temporal resolution than BRET sensors. Intermolecular FRET sensors for GPCR-β-arr interaction have been developed in a similar way to the BRET sensors. The first such sensors used an ECFP donor and an EYFP acceptor fused to the GPCR C-terminus and β-arr C-terminus respectively (Kraft et al., 2001). These FRET constructs indicate β-arr recruitment which occurs after a short lag following receptor activation which corresponds to signal transduction followed by receptor phosphorylation (Krasel et al., 2005). Just like for conformational GPCR biosensors, the large size of the GFP variants used in FRET reporters could disrupt native GCPR-β-arr interaction or downstream signalling, so alternative constructs have been developed with the YFP acceptor replaced with FlAsH by incorporating its tetracysteine binding motif into the C-terminus of β-arr. In a further modification, the CFP donor was also removed and replaced with ReAsH (red arsenical hairpin binder). By incorporating a binding motif with higher affinity for FlAsH into β-arr and a motif with higher affinity for ReAsH into the receptor’s C-terminus, with sequential labelling and washing steps, FlAsH/ReAsH FRET labelling was achieved (Zurn et al., 2010). In cells expressing ReAsH-parathyroid hormone receptor (PTHR), and FlAsH-β-arr, receptor stimulation by parathyroid hormone resulted in a FRET change representing β-arr recruitment (Zurn et al., 2010). The significantly smaller sizes of these fluorophores compared to GFP fluorophores and the reduced steric hinderance makes them more suitable for use when studying downstream β-arr signalling, although their use require a complex labelling protocol in comparison to the CFP/YFP FRET construct, which is completely genetically encoded. Irrespective of whether FRET or BRET is used, one difficulty with intermolecular sensors is ensuring equal expression of both constructs. A construct similar to SPASM in which the GPCR and β-arr are linked in a single construct has not yet been developed but would circumvent this problem.

Another disadvantage of these intramolecular assays is the use of modified receptors with BRET or FRET moieties fused to them which, given the importance of the GPCR C-terminus in downstream signalling, could affect β-arr recruitment. One way of circumventing this is a bystander BRET assay in which rather than the GPCR, one-half of the BRET pair is fused to a membrane-localised protein and thus recruitment of β-arr to the receptor generates a BRET signal due to its localisation in the same compartment (Figure 3B) (Clayton et al., 2014). This approach can be extended by combining the RLuc8 tagged β-arr with a BRET acceptor targeted to different endomembrane compartments by conjugation with different targeting domains (Namkung et al., 2016). These include, for example, the acylation motif of Lyn-kinase for plasma membrane targeting, the FYVE domain for early endosomal targeting and Rab proteins localised to vesicles. These constructs allow the tracking of the progression of β-arr through these compartments following receptor activation. This approach has been developed using an RLuc donor and both citrine and rGFP as the BRET acceptor with the latter having improved sensitivity. Given the non-specific nature of the BRET signal in a bystander-based assay, it is worth noting that BRET signal can be caused by β-arr recruitment to receptors other than those being studied but this setup has the benefit of being useable with any GPCR without the need to generate an RLuc tagged construct.

The two requirements of a receptor for it to recruit β-arr is that it is in its active conformation, and that it is phosphorylated by a GPCR kinase (GRK) (Jean-Charles et al., 2017). The interaction of GPCRs with GRKs has also been studied using BRET-based biosensors (Figure 3C). The first such sensor consisted of an oxytocin receptor with a C-terminally fused RLuc donor and GRK2 tagged also at its C-terminal with a YFP acceptor (Hasbi et al., 2004). This demonstrated an immediate transient agonist-induced BRET increase. When using a similar BRET sensor for β-arr recruitment, the gain of BRET begins after a time lag of around 10 s, presumably corresponding to the recruitment of GRK and receptor phosphorylation. These sensors provide a good platform to study the different selectivities and functions of the seven GRK subtypes. For example, C-terminally RLuc tagged NK-1 receptors, and GRKS 2 and 5 C-terminally fused to GFP have been used to study NK-1 receptors which have been shown to be phosphorylated by both GRK2 and GRK5 (Jorgensen et al., 2008). These experiments demonstrated differences in the dynamics of GRK subtype recruitment to agonist-stimulated receptors. One consideration when using these sensors to investigate GRKs 4, 5, or 6 is that they are membrane bound and so a non-specific BRET signal may arise due to the bystander effect described earlier. Additionally, given the interaction of some GRKs with Gβγ subunits, a BRET signal does not necessarily indicate direct interaction with the receptor itself.

### 4.2 β-arrestin intramolecular conformational biosensors

Whilst the first generation of fluorescent biosensors measured intermolecular RET between the active receptor and β-arr, observations relating to independent signalling of arrestin have led to the development of a second wave of sensors. In addition to the conformation of the active GPCR being important for arrestin recruitment, we now know that arrestins interact with different receptors in different ways. Class B GPCRs bind β-arr with a higher affinity and are internalised as a single macromolecular complex of the phosphorylated GPCR bound to the arrestin. Class A GPCRs, by contrast, bind β-arr transiently, activating the arrestin which then dissociates and remains active for some time during which it likely signals independently (Nuber et al., 2016).

Additionally, structural studies have shown that arrestins predominantly bind to GPCRs in two different conformations. The ‘tail’ conformation in which the arrestin associates only with the receptor’s C-terminus (Thomsen et al., 2016) and the ‘core’ conformation in which the arrestin binds to the receptor’s intracellular core region in addition to the receptor’s C-terminus (Kang et al., 2015). It is likely that these different conformations are responsible for different functional outputs as arrestin bound in the core conformation prevents G-protein coupling whilst arrestin recruitment in the tail conformation does not (Kang et al., 2015). These structural insights have expanded interest somewhat from sensors which can detect a binary interaction between β-arr and a GPCR to probes which can directly indicate β-arr conformation.

The first such sensor was a BRET-based construct consisting of a β-arr molecule with an N-terminally fused RLuc donor and a C-terminally fused YFP acceptor (Figure 3D) (Charest et al., 2005). This probe was tested with receptors from both classes. For class A, the construct was co-expressed with β_2_-AR, V_1_ vasopressin receptors, and δ-opioid receptors. Amongst class B GPCRs, platelet-activating factor receptor (PAFR), CC chemokine receptor type 5 (CCR5), angiotensin II receptor type 1 (AT_1_R), and the vasopressin V_2_ receptor (V_2_R) were used. In all cases, agonist stimulation resulted in redistribution of the sensor to the plasma membrane, representing β-arr recruitment to an active receptor, and a significant and stable increase in BRET, demonstrating conformational rearrangement of the arrestin itself. A smaller BRET change was also consistently reported from the activation of class A GPCRs compared to those in class B. This first intramolecular β-arr BRET construct demonstrates the potential of this system as a general sensor of β-arr recruitment and activation and perhaps to differentiate novel receptors as class A or class B receptors. One benefit of this intramolecular construct compared with an intermolecular design in which the donor and acceptor molecules are fused to separate proteins is that it overcomes the difficulty in ensuring equal expression of the two BRET moieties.

These sensors were further developed by two groups who both independently replaced the BRET acceptor with FlAsH which is not genetically encodable, but has the benefit of being significantly smaller than a fluorescent protein, thus reducing the risk of interference with the conformational change required for signal detection (Lee et al., 2016; Nuber et al., 2016). One group kept an N-terminally tagged RLuc as the BRET donor whilst the other replaced it with a CFP FRET donor and inserted the FlAsH binding motif at different positions within the β-arr sequence. In both cases several constructs were generated which can give a detailed picture of β-arr conformation in live cells in real time. These sensors were used in experiments which demonstrated a cycle of conformational changes by β-arr upon GPCR activation and were the first to show that arrestins remain in an active conformation after dissociation from a receptor providing a possibility of signal amplification by one active receptor binding and activating several arrestins (Nuber et al., 2016). These intramolecular sensors were also used in experiments that demonstrated for the first time a functional relevance of these conformational changes to downstream signalling by revealing that the direction of BRET change consistently predicts downstream β-arr-dependent ERK1/2 activation (Lee et al., 2016). Other similar BRET sensor constructs have been developed which utilise alternative BRET acceptors and NanoLuc as the BRET donor (Oishi et al., 2020) which has the advantage of being smaller and brighter than RLuc. Intramolecular BRET sensors based on β-arr have the potential to identify ligands with β-arr biased signalling whilst intramolecular FRET sensors, with their higher temporal resolution, may be more suitable to study the kinetics of β-arr recruitment and activation.

### 4.3 Split luciferase-based sensors for β-arrestin recruitment

As for G-protein recruitment, NanoBiT-based assays have been developed for recruitment of β-arr (Figure 3E). Using an SmBiT-tagged GPCR, and LgBiT-tagged β-arr, arrestin recruitment is indicated by an increase in luminescence on NanoBiT reconstitution (Dixon et al., 2016; He et al., 2018). These reporters display different luminescence profiles for Class A and Class B GPCRs. The transient recruitment of β-arr to a Class A GPCR such as β2-AR is represented by a fast increase in luminescence intensity followed by a rapid decrease, while the more stable interaction between β-arr and a Class B receptor such as vasopressin V_2_R is indicated by a slower but more sustained increase (Dixon et al., 2016).

The bystander effect discussed in relation to BRET constructs has also been taken advantage of to develop split luciferase assays which can be used with unmodified receptors. In this case, one luciferase fragment is fused to β-arr and a complementary fragment fused to a localising domain (Figure 3F) (Hauge Pedersen et al., 2021). This approach was first attempted using NanoBiT but it failed to produce a functional assay. This was overcome by using NanoLuc split at a different site to NanoBiT into roughly equally sized complementary fragments (N-terminal fragments: amino acids 1–102; C-terminal fragment: amino acids 103–172). The C-terminal fragment was fused to the N-terminus of β-arr, and the N-terminal fragment was fused to either a membrane targeting domain or the FYVE domain to localise it to early endosomes. These constructs have the advantage of being able to measure β-arr recruitment to endogenous receptors without the need for C-terminal modifications. As for all bystander-based assays, the action of off-target receptors must be considered, and a parallel approach used when screening compounds or a knockout model if available.

## 5 Sensors for intracellular second messengers

As discussed in Section 3, the sixteen human Gα subunits fall into four families–Gα_s_, Gα_i/o_, Gα_q/11_, and Gα_12/13_—which each regulates a distinct downstream effector (Wootten et al., 2018). Gα_s_ and Gα_i_ respectively stimulate and inhibit adenylyl cyclase which catalyses the conversion of ATP to the second messenger 3′,5′-cyclic adenosine monophosphate (cAMP) (Levitzki, 1988). Gα_q_ stimulates phospholipase C (PLC) which cleaves phosphatidylinositol 4,5-bisphosphate (PIP2) to diacylglycerol (DAG) and inositol 1,4,5-trisphosphate (IP3). IP3 acts at the endoplasmic reticulum (ER), binding to the IP3 receptor which acts as a calcium channel, allowing extrusion of Ca^2+^ from the ER, raising intracellular calcium (Berridge, 2009). The fourth Gα family–Gα_12/13_—effects downstream signalling via the Rho family of GTPases (Siehler, 2009). Interrogation of these signalling events in live cells in real time has been made possible through the development of a variety of fluorescent biosensors that have contributed to elucidate many novel aspects such as spatiotemporal compartmentalisation of intracellular signalling.

FRET-based biosensors for cAMP and PKA signalling (summarised in Table 4) have contributed greatly to our understanding of this signal transduction cascade downstream of GPCR activation (Lohse et al., 2023) and have paved the way for many of the FRET-reporters developed since for other second messengers and intracellular signalling molecules. We detail their development below as an illustrative example of sensor design and optimisation over many years by different groups. Fluorescent and bioluminescent biosensors for IP3, DAG, and protein kinase C (PKC) are also described in this section. Biosensors for calcium which have largely been used in excitable cells in contexts independent of GPCR signalling are also discussed briefly as they are worth considering for investigations into Gα_q_-stimulated calcium signals.

**TABLE 4:** Sensors discussed in Section 5.1 for detection of intracellular cAMP and PKA activity or phosphorylation.

Fluorescent sensor (use)	Structural details	References
FlCRhR ‘Flicker’ (cAMP sensor, PKA activity)	Fluorescein/rhodamine FRET pair bound to catalytic and regulatory subunits of PKA respectively	Adams et al. (1991)
Genetically encoded PKA-based sensor (cAMP sensor, PKA activity)	CFP/YFP FRET pair bound to regulatory and catalytic subunit of PKA respectively	Zaccolo et al. (2000)
Epac-camps (cAMP sensor)	cyclic nucleotide binding domain (CNBD) from Epac sandwiched between CFP/YFP FRET pair	Nikolaev et al. (2004)
Epac-S^H187^ (cAMP sensor)	Full-length Epac sandwiched between mTurqiose2/circularly permuted (cp)Venus FRET pair	Klarenbeek et al. (2015)
HCN2-camps (cAMP sensor)	CNBD of hyperpolarisation-activated cyclic nucleotide-gated potassium channel HCN2 sandwiched between CFP/YFP FRET pair	Nikolaev et al. (2006a)
ICUE1 (targeted cAMP sensor)	Full-length Epac1 sandwiched between ECFP/citrine FRET pair with targeting domain bound to ECFP or citrine	DiPilato et al. (2004)
CAMYEL (cAMP sensor)	Epac1 mutant sandwiched between RLuc/EYFP BRET pair	Jiang et al. (2007)
ICUE2-based BRET sensor (cAMP sensor)	Epac CNBD sandwiched between RLuc/YFP BRET pair	Barak et al. (2008)
Epac-based BRET^2^ sensor (cAMP sensor)	Epac1 mutant sandwiched between RLuc/GFP BRET pair	Leduc et al. (2009)
cAMP universal tag for imaging experiments (CUTie) (targeted cAMP sensor)	ECFP/EYFP FRET pair fused to C-terminus and loop region of CNBD from PKA-RIIβ. Targeting domain is fused to CNBD N-terminus	Surdo et al. (2017)
A-kinase activity reporter ‘AKAR’ (PKA phosphorylation)	CFP/YFP FRET pair fused to phosphoamino acid binding domain and PKA-specific phosphorylatable sequence	Zhang et al. (2001)
AKAR4 (PKA phosphorylation)	Cerulean/cpVenus FRET pair fused to phosphoamino acid binding domain and PKA-specific phosphorylatable sequence	Depry et al. (2011)

### 5.1 Sensors for cAMP and PKA

Around two-thirds of currently druggable GPCRs signal via Gα_s_ or Gα_i_ proteins and therefore via cAMP (Sriram and Insel, 2018). cAMP signalling is coupled to GPCR activation as the enzyme that generates cAMP–adenylyl cyclase (AC)—is stimulated by active Gα_s_ proteins and inhibited by active Gα_i_ (Levitzki, 1988). cAMP exerts its cellular effects via four known effector proteins: exchange protein-activated by cAMP (Epac), cyclic nucleotide-gated channels (CNGC), Popeye domain-containing proteins (POPDC), and its classical effector protein kinase A (PKA). The specificity of response arising from activation of distinct GPCRs acting via cAMP signalling is now known to be achieved via spatiotemporal compartmentalisation of cAMP signals whereby rather than a homogenous cytosolic rise in cAMP upon Gα_s_ protein activation, there are specific subcellular nanodomains in which cAMP varies (Zaccolo et al., 2021). Our understanding of the contribution of cAMP compartmentalisation to GPCR-mediated signalling is rapidly growing and many of the experiments leading to this understanding have used a variety of fluorescent biosensors which measure either cAMP concentration or PKA activity.

**FIGURE 4:**
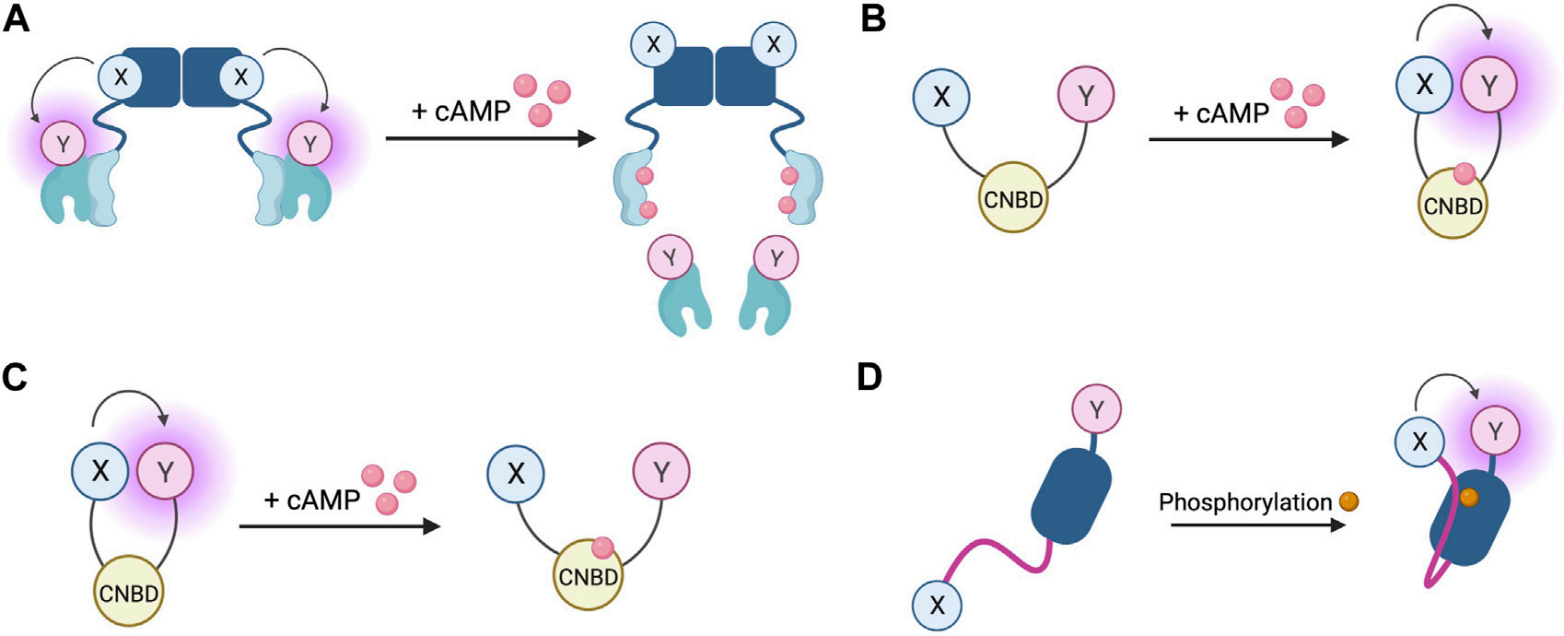
Schematic representations of sensors for cAMP and PKA **(A)** Generic PKA heterotetrameric cAMP FRET sensor with donor fluorophore X bound to PKA regulatory subunit and acceptor fluorophore Y bound to PKA catalytic subunit. X and Y can be a fluorescein/rhodamine FRET pair or YFP/CFP FRET pair **(B)** Generic gain of FRET cAMP sensor with donor and acceptor fluorophores X and Y bound to cyclic nucleotide binding domain (CNBD). See Table 4 for examples of CNBD and fluorophores. **(C)** Generic loss of FRET cAMP sensor with donor and acceptor fluorophores X and Y bound to cyclic nucleotide binding domain (CNBD). See Table 4 for examples of CNBD and fluorophores. **(D)** PKA activity reporter (AKAR) with donor and acceptor fluorophores X and Y bound to phosphoamino acid binding domain (dark blue) and PKA-specific phosphorylatable sequence (purple line). See Table 4 for example, fluorophores.

The first fluorescent sensors for cAMP were developed in 1991 and exploited the heterotetrametric structure of PKA (Adams et al., 1991). Recombinant regulatory and catalytic subunits of PKA were expressed in *E. coli* and, after purification, they were tagged with the fluorophores rhodamine and fluorescein, respectively and then transferred by microinjection into living cells. The PKA subunits labelled with the fluorophores can still combine to form a functioning holoenzyme with two catalytic and two regulatory subunits. Given the proximity of the fluorophores, upon excitation, fluorescein transfers energy by FRET to the rhodamine acceptor. The emission from rhodamine can then be detected. Upon binding of cAMP to PKA, the holoenzyme dissociates to allow activation of the catalytic subunits and the distance between the fluorophores increases to infinity, abolishing FRET and causing a detectable change in the ratio between rhodamine and fluorescein fluorescence (Figure 4A) (Adams et al., 1991). To reflect the structure of these sensors with fluoresceine bound to the catalytic subunit and the rhodamine bound to the regulatory subunit, they were named FlCRhR (pronounced ‘flicker’). Not all cells are amenable to microinjection, so this technique has been largely applied to non-excitable or large excitable cells such as giant neurons (Liu et al., 1999). To use this sensor in small excitable cells, a protocol allowing passive diffusion of the sensor into cells from a patch pipette has been used (Goaillard et al., 2001).

In order to allow this system to be generalisable to all cell types and to overcome the technical challenge of having to microinject the purified sensor into cells, a genetically encoded FRET sensor was developed that still relies on dissociation of the PKA heterotetramer upon cAMP binding, but where the fluorescein and rhodamine fluorophores were replaced with a CFP donor and YFP acceptor (Zaccolo et al., 2000). These sensors were used in the first experiments to demonstrate that stimulation of β-AR generates multiple distinct subcellular pools of cAMP and that such compartmentalisation of cAMP to form spatially confined gradients is regulated by phosphodiesterases (PDEs) (Zaccolo and Pozzan, 2002). Although easier to use than FlCRhR sensors, these genetically encoded sensors do share some common disadvantages. Due the intermolecular nature of the constructs, ensuring similar levels of expression of the acceptor and donor fluorophores is not guaranteed and this problem is further exacerbated by the possibility of the fluorescent constructs assembling into holoenzymes with endogenous PKA subunits, reducing the number of FRET-competent holoenzymes. Furthermore, the cooperative nature of four cAMP molecules binding to a single PKA holoenzyme means the slow kinetics of the FRET change may not fully represent an intracellular rise in cAMP (Nikolaev et al., 2004). Despite these limitations, the PKA-based FRET reporters are the only sensors to date which directly measure PKA activation, so may be suitable for experiments where this aspect is of interest (Koschinski and Zaccolo, 2017).

Circumvention of the problems posed by intermolecular sensors has been achieved using intramolecular FRET sensors in which the FRET donor and acceptor are both fused to the same molecule (Figures 4B, C). Such sensors have been developed using cyclic nucleotide binding domains (CNBD) originating from a variety of endogenous cAMP effector proteins. Many of these sensors derive their CNBD from exchange protein-activated by cAMP (Epac). The Epac protein family consists of Epac1 and Epac2 which are encoded by two independent genes in mammals (Cheng et al., 2008). Epac functions as a guanine nucleotide exchange factor (GEF) for Ras family proteins Rap1 and Rap2 and is activated by cAMP binding (de Rooij et al., 1998; Kawasaki et al., 1998). The latest iterations of Epac-based FRET sensors are some that provide the largest dynamic ranges of up to 150% (Klarenbeek et al., 2015). Two of the most popular Epac-based sensors are Epac1-camps (Nikolaev et al., 2004) and EPAC-S^H187^ (Klarenbeek et al., 2015). The large dynamic range of the last generation of the Epac-based sensors makes them ideal for experiments where detection of small differences in cAMP signalling are important. Both the PKA- and the Epac-based sensors have EC50 for response to cAMP in the range of approximately 1–3 μM, which makes them suitable to detect physiological concentrations of the second messenger in most cell types (Nikolaev et al., 2004; Klarenbeek et al., 2015; Koschinski and Zaccolo, 2017; Surdo et al., 2017). HCN2-camps - a sensor which instead uses the CNBD from the hyperpolarisation-activated, cyclic nucleotide-gated potassium channel HCN2 with a FRET acceptor and donor fluorophore at each terminus is more suited to measure high levels of cAMP due to its lower sensitivity (Nikolaev et al., 2006a). The Epac and HCN sensors have been used to characterise cytosolic cAMP responses to a variety of agonists in both health and disease, for example, demonstrating that β1 and β2 adrenergic receptors elicit different spatial patterns of cAMP signalling and that this difference is diminished in heart failure (Nikolaev et al., 2006a; Nikolaev et al., 2010). Epac-based sensors are more suited to bulk cytosolic measurement whereas the PKA sensors discussed earlier are largely localised to A-kinase anchoring proteins (AKAPs) and report cAMP signals from localised signalling nodes (Zaccolo and Pozzan, 2002).

In addition to Epac-based FRET sensors, cAMP BRET sensors have been developed using Epac as their CNBD. CAMYEL consists of an inactive mutant of Epac1 sandwiched between an RLuc and eYFP BRET pair (Jiang et al., 2007). This has been further developed for improved brightness by swapping the eYFP BRET acceptor for GFP (Leduc et al., 2009). The ICUE2 FRET sensor which uses an N-terminal truncated version of Epac as its CNBD, has also been adapted into a BRET sensor with an N-terminal RLuc donor and C-terminal YFP acceptor (Barak et al., 2008). The advantage of a cAMP BRET *versus* a FRET reporter is its suitability to high throughput screening as it can be used in a 96-well plate with a standard plate reader.

In order to better interrogate compartmentalisation of cAMP signalling targeted sensors have been developed which allow measurement of cAMP at distinct subcellular locations. Targeted ICUE1 sensors were developed using the CAAX-box sequence to localise the sensor to the plasma membrane, a nuclear localisation sequence to retain the sensor in the nucleus, or a sequence from cytochrome-C oxidase to localise the sensor to mitochondria (DiPilato et al., 2004). These targeted sensors allow measurement of differential dynamics of cAMP responses at each of these subcellular locations, demonstrating that the cAMP response is fastest at the plasma membrane, followed by the cytosol and mitochondria and slowest within the nucleus (DiPilato et al., 2004). One shortfall of these constructs is that the targeting domain is fused directly to one of the fluorophores, meaning that depending on the size and structure of each specific targeting domain and the consequent steric hindrance on the fluorophore it is fused to, the conformational change that takes place on cAMP binding may be affected which adds a degree of uncertainty to direct comparisons between differently targeted sensors. cAMP universal Tag for Imaging Experiments (CUTie) was designed specifically to avoid this problem (Surdo et al., 2017). CUTie uses the CNBD from PKA-RIIβ regulatory subunit but unlike all of the cAMP FRET sensors discussed so far, the fluorophores are not fused at the N- and C-termini. Instead, the ECFP donor is bound to the C-terminus of the CNBD and the EYFP acceptor is incorporated into an external loop-region of the CNBD. This allows the targeting domain to be fused to the CNBD directly, distally to the EYFP rather than to the fluorophore itself as in targeted EPAC and HCN sensors. This allows comparability between the different localised CUTie sensors as any differences in steric hinderance are minimised. CUTie sensors have been used extensively in cardiac myocytes and constructs have been generated which localise to the plasma membrane, sarcoplasmic reticulum (SR), contractile filaments, and several PKA scaffolding proteins (Surdo et al., 2017).

In addition to cAMP sensors, FRET constructs have been developed to measure PKA activity. A-kinase activity reporters (AKARs), are four-part chimeric proteins consisting of a CFP FRET donor, a phosphoamino acid binding domain, a PKA-specific phosphorylatable sequence, and a YFP FRET acceptor (Zhang et al., 2001). When PKA phosphorylates the phosphorylatable sequence, the construct undergoes rearrangement as the phosphoamino acid binding domain forms an intramolecular complex with the phosphorylated peptide. This conformational change reduces the distance between the fluorophores, resulting in a gain of FRET (Figure 4D). Localised AKAR constructs have also been developed, targeting the sensor to the nucleus with a nuclear localisation sequence or tethering the sensor to AKAPs to co-localise the activity reporter with PKA (Zhang et al., 2001). The latest iteration of AKAR (AKAR4) which uses brighter fluorophores cerulean as its FRET donor and circularly permuted YFP variant cpVenus as its FRET acceptor has a much-improved dynamic range (Depry et al., 2011). Plasma membrane targeted AKAR constructs, which localise to lipid rafts or non-raft regions of the membrane, have been developed and used to explore PKA dynamics in different membrane microdomains and demonstrate the role of lipid rafts in regulating PKA activity at the membrane (Depry et al., 2011). One aspect that must be taken into account when using AKAR4 to measure PKA activity is that, in addition to being phosphorylated by PKA, AKAR4 can be dephosphorylated by phosphatases and so the detected signal represents a balance between PKA and phosphatase activity.

Targeted FRET reporters for cAMP and PKA have been used widely to characterise the spatiotemporal compartmentalisation of cAMP signalling. One innovative set of experiments using these sensors made use of constructs in which the cAMP FRET sensor Epac1-camps is directly conjugated to the c-terminus of a glucagon-like peptide receptor (GLP-1) (Anton et al., 2022) to show that low (picomolar) concentrations of GLP1 exclusively generate a GLP-1R-associated cAMP pool which does not evoke a cAMP response in the bulk cytosol or at other locations of the plasma membrane at a distance from this receptor. Using single-alpha-helical domain (SAH) linkers as ‘nanorulers’ of defined lengths placed between the FRET sensor and the receptor, the size of these plasma membrane cAMP nanodomains was able to be determined. These nanodomains were termed ‘receptor associated independent nanodomains’ or RAINs (Anton et al., 2022). This is one example of how these sensors have been used to refine our understanding of cAMP compartmentalisation and the complexity of GPCR signalling.

### 5.2 Sensors for IP3, DAG, and PKC

**FIGURE 5:**
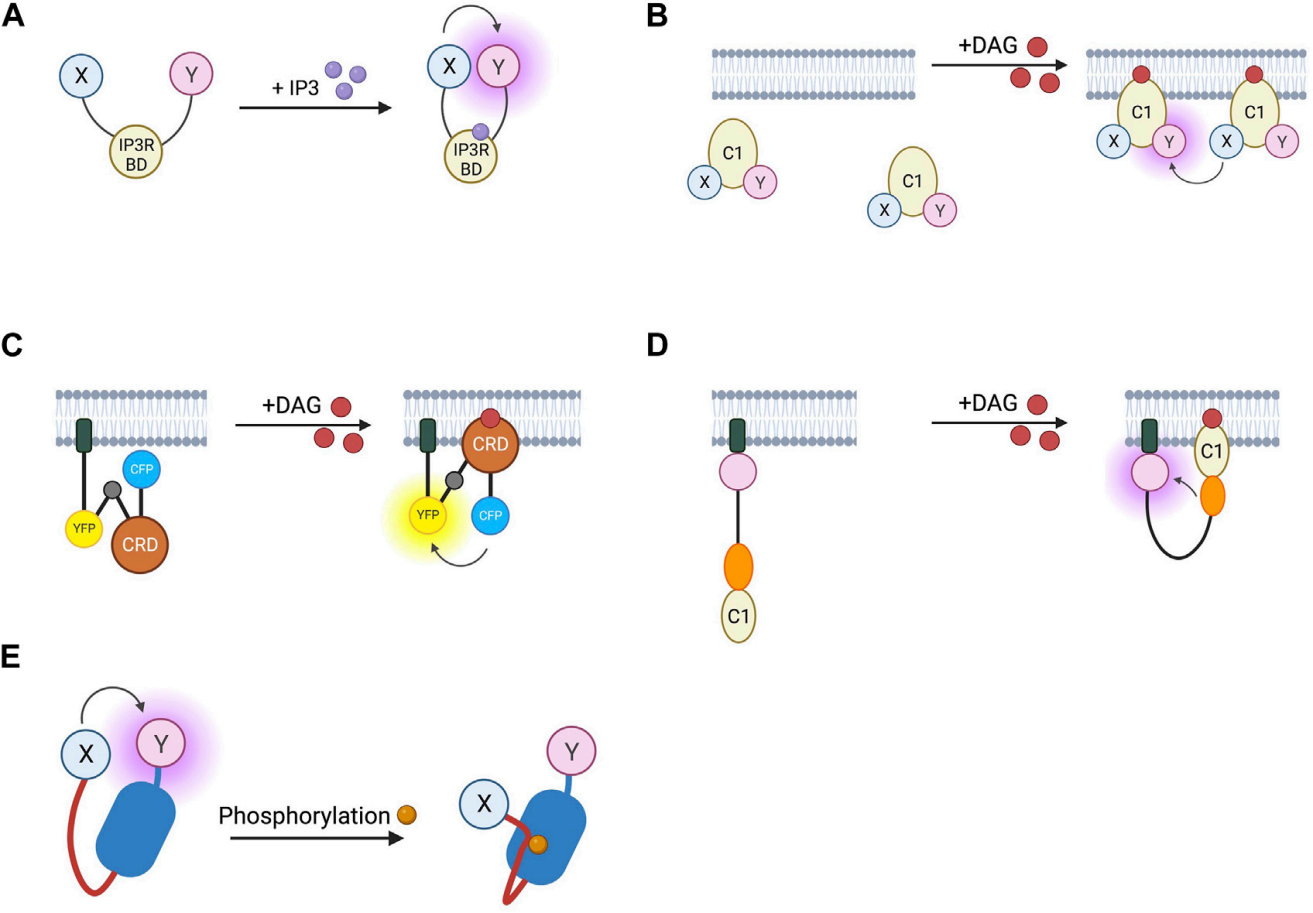
Schematic representations of sensors for IP3, DAG, and PKC **(A)** IP3 RET sensor with energy donor and acceptor X and Y fused to IP3 binding domain from IP3 receptor. **(B)** DAGR FRET sensor for DAG with donor and acceptor fluorophore X and Y fused to C1 DAG binding domain. **(C)** Daglas FRET sensor for DAG consisting from N- to C-terminal, of a membrane anchor, α-helical linker, YFP FRET acceptor, α-helical linker with Gly-Gly hinge (grey circle), cysteine rich domain or DAG binding, α-helical linker, and CFP FRET donor. **(D)** Unimolecular DAG BRET sensor consisting from N- to C-terminus of a membrane anchor, GFP BRET acceptor, unstructured linker, RLuc BRET donor, and C1b DAG binding domain. **(E)** PKC activity reporter (CKAR) with energy donor and acceptor X and Y fused to phosphothreonine binding domain (dark blue) and PKC-specific phosphorylatable sequence (red line).

IP3 and DAG are produced by the cleavage of PIP2 by phospholipase-Cβ and are the second messengers which mediate signal transduction of Gα_q_-coupled GPCRs. IP3 acts at intracellular IP3 receptors (IP3R) to release intracellularly stored Ca^2+^ whilst DAG recruits protein kinase C (PKC) to the membrane where the kinase is activated (Neves et al., 2002). FRET-based IP3 reporters (summarised in Table 5) work in much the same way as cAMP FRET sensors, with an IP3 binding domain taken from the IP3R sandwiched between a FRET donor and acceptor (Figure 5A). The first of these sensors to be developed were the LIBRA sensors which use a CFP/YFP FRET pair and are targeted to the cell membrane with a fragment of neuromodulin or, in later iterations, with GAP43 (Tanimura et al., 2004; Tanimura et al., 2009). Further improvements to later versions of these sensors include the replacement of YFP with Venus to reduce pH sensitivity, and producing a spectrum of sensors with differing IP3 affinities by varying the IP3 binding domain (Tanimura, 2011). Similar constructs use alternative FRET pairs such as EGFP and Halo-protein with tetramethylrhodamine as donor and acceptor respectively (Matsu-Ura et al., 2019).

**TABLE 5:** Sensors discussed in Section 5.2 for detection of intracellular IP3 and DAG, and PKC activity.

GPCR signalling step	Detection method/mechanism	References
IP3 generation	CFP/YFP FRET	Tanimura et al. (2004), Tanimura et al. (2009)
	EGFP/tetramethylrhodamine FRET	Matsu-Ura et al. (2019)
DAG generation	CFP/YFP FRET “DAGR”	Violin et al. (2003)
	CFP/YFP FRET “Daglas”	Sato et al. (2006)
	RLuc/GFP BRET	Namkung et al. (2018)
PKC activity	CFP/YFP FRET “CKAR”	Violin et al. (2003)
	RLuc/GFP BRET	Namkung et al. (2018)

Biosensors for intracellular DAG have typically used the C1 domain of PKC as the DAG-binding motif. DAG reporter DAGR is composed of a C1 domain tagged at opposite ends with a CFP/YFP FRET pair (Violin et al., 2003). The measurement relies on intermolecular FRET between these constructs rather than FRET changes resulting from a conformational change. At baseline, there is a lower FRET signal as the construct diffuses in the cytosol, upon production of DAG, the construct is recruited to the membrane resulting in a higher effective concentration and therefore an increase in intermolecular FRET (Figure 5B). Given the low signal to noise ratio from intramolecular and intermolecular FRET being indistinguishable, a sensor which relies only on the former has been developed to avoid this. The DAG reporter Daglas also uses a cysteine-rich domain from PKC as its DAG-binding motif (Sato et al., 2006). This is fused via rigid α-helical linkers to CFP and YFP and anchored to the membrane with a membrane localisation sequence. One of the α-helical linkers includes a hinge motif which upon DAG binding to the binding domain results in sufficient conformational rearrangement for a gain of FRET (Figure 5C). More recently, a BRET-based DAG sensor has been developed which is analogous to the BERKY discussed earlier (Namkung et al., 2018). From N- to C-terminus, it consists of a membrane localisation sequence, GFP BRET acceptor, a 300 amino acid unstructured linker, RLuc BRET donor, and the c1b DAG binding domain of PKC. Upon generation of DAG, the linker bends, resulting in decreased distance between the BRET pair and an increase in the BRET signal (Figure 5D). These constructs were shown to respond to DAG generation from Gα_q_-coupled receptors including angiotensin II AT_1_R and muscarinic M3 receptors and represent the kinetics of Gα_q_ activation with an initial BRET increase over 30 s, followed by a return to baseline over 5 min.

Activation of PKC following Gα_q_-mediated signalling has also been interrogated with RET sensors. CKAR is analogous to the PKA activity reporter AKAR and consists of a CFP/YFP FRET pair linked by a PKC phosphorylatable sequence and an FHA2 phosphothreonine binding domain (Violin et al., 2003). Phosphorylation of the PKC substrate leads to an intramolecular interaction with the FHA2 domain and the resulting conformational change leads to a decrease in FRET (Figure 5E). A BRET construct based on the same backbone with an RLuc/GFP BRET pair has also been developed as an intracellular PKC reporter (Namkung et al., 2018). These reporters have an addition of the C1b DAG binding domain which tethers the reporter via DAG to the membrane in the proximity of Gα_q_-coupled receptors, thus localising the sensor in the proximity of PKC isoforms activated by Gα_q_-signalling.

### 5.3 Sensors for intracellular Ca^2+^

Intracellular calcium signalling is often investigated using synthetic calcium dyes. While these are beyond the scope of this review they are reviewed excellently elsewhere (Li and Saha, 2021; Lohr et al., 2021). There is also a wide variety of genetically encoded calcium indicators (GECIs) available which are either single wavelength or FRET-based (summarised in Table 6).

**TABLE 6:** Sensors discussed in Section 5.3 for detection of intracellular calcium.

Sensor	Structural details	References
Pericam	Circularly permutted EYFP (cpYFP) sandwiched between calmodulin (CaM) and CaM-binding peptide M13	Nagai et al. (2001)
G-CaMP	cpGFP sandwiched between CaM and M13	Nakai et al. (2001)
Cameleon	CaM and M13 sandwiched between CFP/YFP FRET pair	Miyawaki et al. (1997), Miyawaki et al. (1999)
Troponin C-based sensor	CFP/YFP FRET pair fused to Troponin C Ca^2+^-binding domain	Heim and Griesbeck (2004)

**FIGURE 6:**
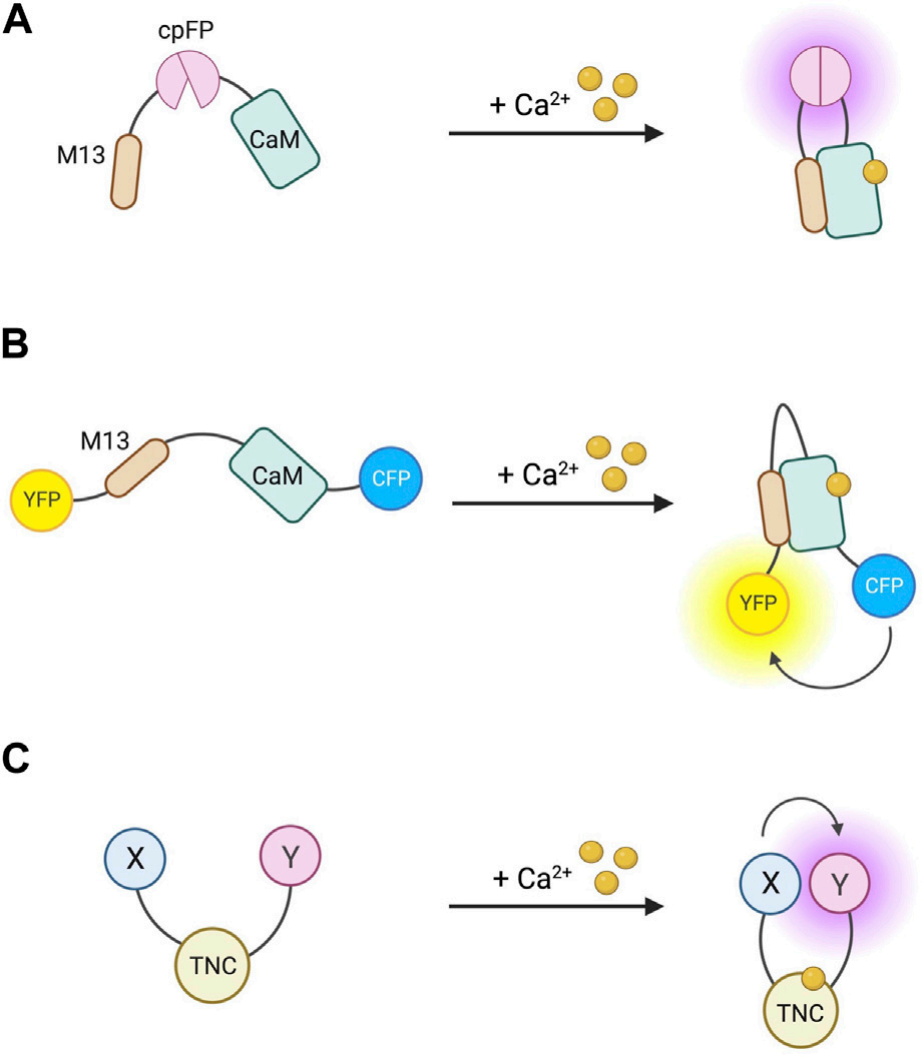
Schematic representations of sensors for intracellular Ca^2+^ **(A)** Pericam/G-CaMP single wavelength calcium indicator with circularly permuted fluorophore (cpFP) sandwiched between calmodulin (CaM) and CaM-binding peptide M13. **(B)** Cameleon Ca^2+^ FRET reporter with CaM and M13 sandwiched between CFP FRET donor and YFP FRET acceptor. **(C)** FRET reporter for Ca^2+^ with donor and acceptor fluorophores bound to troponin C (TNC).

Single wavelength GECIs consist of a fluorescent protein fused to a calcium binding domain which interacts with intracellular Ca^2+^ resulting in a change in the spectral properties of the fluorescent protein, and typically a change in fluorescence intensity. Popular non-ratiometric GECIs include Pericam and G-CaMP. Pericam consists of a circularly permuted EYFP with an N-terminal M13 and C-terminal calmodulin (CaM) (Nagai et al., 2001). CaM is a calcium binding protein and M13 is a CaM binding peptide derived from myosin light chain kinase. Upon binding calcium, the Ca^2+^/CaM binds to M13, resulting in a detectable increase in fluorescence intensity (Figure 6A). This original reporter has been developed into the newer calcium indicators Flash Pericam which has a greater dynamic range, and Inverse Pericam which undergoes a decrease in fluorescence intensity upon calcium binding. GCaMPs were developed at a similar time and are structurally similar but use cpGFP rather than cpEYFP as their fluorophore (Nakai et al., 2001).

Cameleons were the first FRET-based GECIs and similar to Pericams and G-CaMPs, their calcium sensitivity is imparted by CaM and M13 (Miyawaki et al., 1997; Miyawaki et al., 1999). This chimera is sandwiched between a CFP/YFP FRET pair which upon calcium binding leads to a gain of FRET (Figure 6B). One predicted source of uncertainty when using these sensors is that CaM is endogenously highly expressed and can interact with many different partners, resulting in changes in FRET. FRET sensors without the CaM/M13 construct would avoid this issue, for example, Ca^2+^ FRET reporters based on troponin C (Figure 6C) (Heim and Griesbeck, 2004). The single-wavelength and FRET-based reporters have different advantages and disadvantages, making them better suited to different experiments. FRET sensors are more appropriate for determining intracellular Ca^2+^ concentration, whilst non-ratiometric reporters may be better suited to studying Ca^2+^ dynamics.

## 6 Discussion

The use of light-emitting biosensors has greatly enhanced our understanding of GPCR signalling, elucidating novel aspects, including biased agonism and spatiotemporal compartmentalisation of intracellular signals.

Intermolecular constructs making use of fluorescence and bioluminescence resonance energy transfer (FRET and BRET) and circularly permuted fluorescent proteins (cpFP) have provided insight into GPCR activation. For example, they have been instrumental in revealing the existence of multiple active conformations of individual receptors and have provided data in support of the notion that different ligands differentially stabilise specific active conformations (Section 2.1), providing a possible mechanism for partial and inverse agonism as well as a basis for ligand-bias. Adapting these biosensors for use in high-throughput screening could be useful in future to screen drug candidates for activity at GPCRs and categorise them as full, partial, or inverse agonists, or as antagonists. It is clear that some drugs currently in clinical use may have been miscategorised. For example, data show some beta-blockers are able to stimulate β-AR to some degree (Barrett and Carter, 1970; Benkel et al., 2022), suggesting their classical description as antagonists of these receptors may be incorrect, and they may be better classified as partial agonists. Having a robust assay to determine the action of drugs on GPCRs in live cells would facilitate a better mechanistic understanding of drugs currently in use, allowing them to be improved more quickly.

Fluorescent heavy-chain only antibodies or ‘nanobodies’ which bind specifically to active-conformation receptors have elucidated the existence of active GPCRs on internal membranes, including on endosomes and the Golgi (Section 2.2). Experimental data have already demonstrated potential clinical applications of targeting GPCRs at internal membranes for analgesia, as specific antagonism of neurokinin 1 receptors on endosomes elicited more effective pain relief than conventional membrane targeted antagonists (Jensen et al., 2017). Further work is needed to understand the different roles of this receptor pool to those at the cell surface and the tools discussed in this review will likely be of great use.

Furthermore, fluorescent and bioluminescent biosensors have refined our ability to measure G-protein coupling to receptors and G-protein activation (Section 3), revealing that some receptors can couple to multiple G-protein families and that from a single ligand binding event, one receptor may couple to, and activate multiple G-protein heterotrimers. Similar constructs have been developed as inter- and intramolecular β-arr biosensors (Section 4) which have contributed further evidence to the narrative that far from being a simple off-switch, arrestins themselves have an activation cycle and can couple to G-proteins in multiple active conformations which may have roles in G-protein-independent downstream signalling.

Finally, research into second messenger signalling downstream of GPCRs has benefitted hugely from the use of light-emitting biosensors for cAMP, PKA, DAG, IP3, PKC, and Ca^2+^. Our understanding of cAMP and PKA activity, for example, has been advanced tremendously by the development of FRET-based cAMP sensors and PKA activity reporters, revealing subcellular cAMP nanodomains differentially activated by different receptors. This spatiotemporal compartmentalisation of cAMP signalling has gone some way to answer longstanding questions relating to how GPCRs achieve specific responses despite all acting through a narrow range of signalling molecules. Furthermore, a more complete understanding of compartmentalisation of signalling will pave the way for more targeted therapeutics. One illustrative example is milrinone, a non-isoform specific phosphodiesterase 3 (PDE3)-selective inhibitor, which is indicated for the treatment of acute, refractory heart failure, and leads to improvement in symptoms in the short-term but a long-term increase in mortality (Packer et al., 1991). Using an isoform specific PDE3 inhibitor may avoid this problem (Subramaniam et al., 2023) and using the tools outlined in this review during the drug discovery process to determine the effect on specific cAMP nanodomains could minimise similar unanticipated off-target effects in the future.

Fluorescent and bioluminescent biosensors have a wide range of applications for studying GPCR signalling and are suitable to be generalised as high throughput assays which can be implemented in the drug discovery pipeline to reduce the elevated attrition rate of drug candidates. Given the vast number of drugs both on the market and in development which target GPCRs (Sriram and Insel, 2018), deepening our understanding of novel aspects of GPCR signalling will be critical for developing new and more effective GPCR-targeted therapeutics.
